# BreathCarer: Informal carers of patients with chronic breathlessness: a mixed-methods systematic review of burden, needs, coping, and support interventions

**DOI:** 10.1186/s12904-025-01670-0

**Published:** 2025-02-01

**Authors:** Saskia Blütgen, Anne Pralong, Carolin Wilharm, Yvonne Eisenmann, Raymond Voltz, Steffen T. Simon

**Affiliations:** 1 Department of Palliative Medicine, Faculty of Medicine and University Hospital, University of Cologne, Kerpener Street 62, 50937 Cologne, Germany; 2 Faculty of Medicine and University Hospital, Centre for Integrated Oncology, University of Cologne, Aachen-Bonn-Cologne-Duesseldorf (CIO ABCD), Cologne, Germany

**Keywords:** Breathlessness, Chronic, Informal carer, Burden, Needs, Palliative

## Abstract

**Background:**

Breathlessness is a common symptom in chronic and advanced diseases, and it poses a significant burden to patients and to their informal carers. They play a crucial role in sustainable care for patients living with breathlessness, but their challenges and needs are often neglected.

**Objective:**

To provide a systematic overview of the literature on the burden, needs, coping and use of healthcare and social services by carers of patients suffering from chronic breathlessness due to any life-limiting disease.

**Design:**

A mixed-methods systematic review (PROSPERO CRD42022312989).

**Data sources:**

Medline, CENTRAL, PsycINFO, and CINAHL were searched and complemented with forward and backward searches and expert consultation.

**Review methods:**

The mixed-methods review included any study on burden, needs and coping among carers of patients with breathlessness published from the inception of the databases until July 2023. A narrative analysis of the quantitative results and a pragmatic meta-aggregation of the qualitative findings were performed, followed by a mixed-methods convergent segregated approach.

**Findings:**

A total of 53 studies with 4,849 carers were included. Breathlessness is highly burdensome for carers who live with and care for patients or for those who do not live with the patients and care for them. Breathlessness is a significant risk factor for high carers’ burden, contributing to deteriorating physical and mental health among carers and creating an urgent need for external support. A major challenge is the sense of being trapped in a state of constant alertness and anxiety, centred around managing the patient's breathlessness. Carers bear substantial emotional burden due to uncertainty, sleep disturbances, and social isolation, which leads to severe psychological distress. Their unmet needs for professional guidance, self-management strategies, and social interaction are high. While supportive interventions, such as specialized services and multidisciplinary approaches, can alleviate some of the burden, there remains a lack of targeted interventions specifically designed for carers.

**Conclusions:**

This review highlights the substantial burden associated with caring for patients with chronic breathlessness, the unmet needs of carers and the lack of supportive care structures, leaving them with little option but to accept the situation.

**Supplementary Information:**

The online version contains supplementary material available at 10.1186/s12904-025-01670-0.

## What is already known on this topic

Breathlessness is a common and distressing symptom in advanced diseases. Carers play a crucial role in overall patient care, yet being a carer has negative impact on their wellbeing and physical and mental health.

## What this study adds

This qualitative and quantitative overview of existing evidence and literature on carers of patients with breathlessness reveals a deeper understanding of the emotional burden on carers, particularly the constant state of alertness and anxiety caused by breathlessness. It also brings new insights by emphasizing the linkage between carers’ unmet needs and increased breathlessness in patients, highlighting the urgent need for targeted interventions.

## How this study might affect research, practice and/or policy

To support carers of patients with breathlessness, it is essential to strengthen community networks, promote societal recognition, and provide holistic support. Healthcare professionals should address carers’ burdens, offer clear guidance, and teach techniques to manage breathlessness, boosting carers’ confidence and well-being. Research should focus on non-pharmacological treatments, carer-specific interventions, and standardizing terminology with separate patient and carers’ data for better study comparisons.

## Background

The American Thoracic Society (ATS) defines breathlessness as a multifactorial and “subjective experience of breathing discomfort that consists of qualitatively distinct sensations” [1, 2]. Chronic breathlessness syndrome occurs when breathlessness “persists despite optimal treatment of the underlying pathophysiology and that results in disability” [3]. Breathlessness is a common symptom affecting approximately 10% of the global population, worldwide [4]. For instance, in Germany, an estimated 7.4 million individuals experience this condition [5].

Female and older patients are more likely to experience breathlessness [4], and breathlessness is acknowledged as a common and distressing symptom for patients with life-limiting diseases like cancer, chronic heart failure (CHF) or chronic obstructive pulmonary disease (COPD) [6–8]. Experiences of breathlessness—especially episodic breathlessness—are often associated with anxiety [8, 9], leading to a vicious circle in which breathlessness triggers anxiety and anxiety increases breathlessness [10]; this cycle can even lead to fear of death by suffocation [9, 11].

Most patients with chronic breathlessness and life-limiting diseases are dependent on informal carers. We define any person who provides physical, emotional or practical care and support to a relative or friend as an informal carer. In the following, they are referred to as carers. The close relationship between carers and patients enhances how both caregivers and care recipients are affected by a chronic illness, rather than the pure affectedness of patients as individuals [12]. Thus, breathlessness might also be burdensome for carers who live with the patient and care for them [13].

In recent years, the question of the needs and burden of carers supporting patients with chronic breathlessness has become an increasingly common research subject [14, 15]. Irrespective of the diagnosis, the management of chronic breathlessness, which includes pharmacological and non-pharmacological strategies [16], is complex and insufficiently addressed in research, not only with regard to patients but also to their carers [17]. Therefore, carers play a key role in the healthcare management of patients with breathlessness and enable home care [18]. A few systematic reviews exist, but these have a limited focus on qualitative studies only [19], a specific underlying disease [20] or recent advances, thus limiting the systematic literature search to a short period of time [15]. According to preliminary searches and the International Breathlessness Research Group, a systematic search and evaluation of the existing literature on carers for breathless patients is missing.

Therefore, this systematic review aims to identify, appraise and synthesise all qualitative and quantitative evidence on burden, needs, coping and the use of healthcare and social service by carers of patients suffering from chronic breathlessness due to any life-limiting disease. Thus, our review questions were (1) what is the evidence on carers’ burden, (unmet) needs, and coping strategies? And (2) what types of support services do they use and need?

## Methods

This systematic review was conducted according to the Joanna Briggs Institute (JBI) methodology for mixed methods systematic reviews (MMSRs) [21] and is reported using the preferred reporting items for systematic reviews and meta-analyses (PRISMA) [22]. It was registered in advance on the International Prospective Register of Systematic Reviews (PROSPERO), Registration Number: CRD42022312989; the registration includes the protocol.

### Search strategy

The electronic search was performed from the databases inception until July 2023 in four databases: MEDLINE (Ovid), PsycINFO (EBSCOhost), CINAHL (EBSCOhost) and Cochrane Central Register of Controlled Trials (CENTRAL). We created a preliminary search strategy in MEDLINE (Ovid) and tailored syntax and vocabulary to each database. A specialist librarian from the Cochrane Cancer Collaboration reviewed the final search strategies. We combined MeSH terms and keywords with truncation related to carers and breathlessness (see Supplement I). Strategy sensitivity (recall) was tested by means of ‘sentinel papers’, which are already known publications that are expected to be retrieved by the search strategy. We also searched for planned or ongoing studies in the trial registers www.clinicaltrials.gov, International Clinical Trials Registry Platform (ICTRP, https://www.who.int/ictrp/en/) and ISCRTN-Registry (https://www.isrctn.com/) until July 2023. We aimed to identify current research projects that might complement our findings in the near future. The search was complemented by a manual search via checking the reference lists of all included studies and citation tracking over the PubMed filters ‘cited by’ and ‘similar articles’. Furthermore, experts in the field were contacted via e-mail. A final update of the electronic and manual search was made in July 2023.

### Study selection

We used EndNote 20 (Clarivate Analytics, PA, USA) as citation management system and removed duplicates as our first step. Three independent reviewers (SB, AP or StS) screened titles and abstracts against the inclusion criteria (Table 1). The full texts of potential studies were independently screened by three reviewers (SB, AP or CW). Any disagreements between the reviewers at any stage were resolved through discussion with a third reviewer (StS or AP).

**Table 1:** Inclusion criteria

Review questions	Effectiveness and effect of interventions	Description of burden, needs, coping and healthcare/social service use of informal carers	Perception and experience of informal carers
**Inclusion criteria**			
** Study design:**	**Quantitative studies**		**Qualitative studies**
	**Analytic**^a^	**Descriptive**	
	Experimental studies^a^ Observational studies^a^	Descriptive studies^b^ (original research)	Qualitative studies (original research)
**PICO**^c^** / PICo**^d^**:**			
** P**opulation	Informal carers of adult patients with chronic breathlessness due to cancer, COPD, CHF, ILD/IPF, MND^e^ *We define an informal carer as a person of any age providing physical, emotional or practical care and support to a relative or a friend, outside of the context of his/her professional work* Studies with mixed populations (patients and carers) were included if specific results for carers could be extracted		
** I**ntervention/ Topic of **I**nterest	Any intervention aiming to support, inform or train carers	Any quantitative description of burden, needs and coping of carers	Perception and experience of carers in caring or supporting patients
** C**omparator	In studies with control group: standard care or any other intervention	(not applicable)	(not applicable)
** O**utcome	Any outcome for carers specifically related to breathlessness, such as well-being, coping/mastery, knowledge, skills and health care/social service use	Any quantitative descriptive parameter on carers specifically related to breathlessness: prevalence, incidence, experience, etc	(not applicable)
** Co**ntext	Any setting in any country		

^a^ In our understanding, analytical studies are studies attempting to quantify the relationship between the effect of an intervention and an outcome. If the researcher actively imposes an intervention, this is an experimental study. If he/she simply observes the effect of the exposure, this is an observational study, e.g., a cohort or a case–control study (based on CEBM, The Centre for Evidence-Based Medicine, University of Oxford, https://www.cebm.net/2014/04/study-designs/)

^b^ E.g., surveys, case series or case reports

^c^ PICO (Population, Intervention, Comparator, Outcome) refers to four categories of inclusion criteria for quantitative (analytical) studies

^d^ PICo (Population, topic of Interest, Context) applies for qualitative studies

^e^ COPD (chronic obstructive pulmonary disease), CHF (chronic heart failure), ILD (interstitial lung disease)/IPF (idiopathic pulmonary fibrosis), MND (motor neurone disease)

**Fig. 1:**
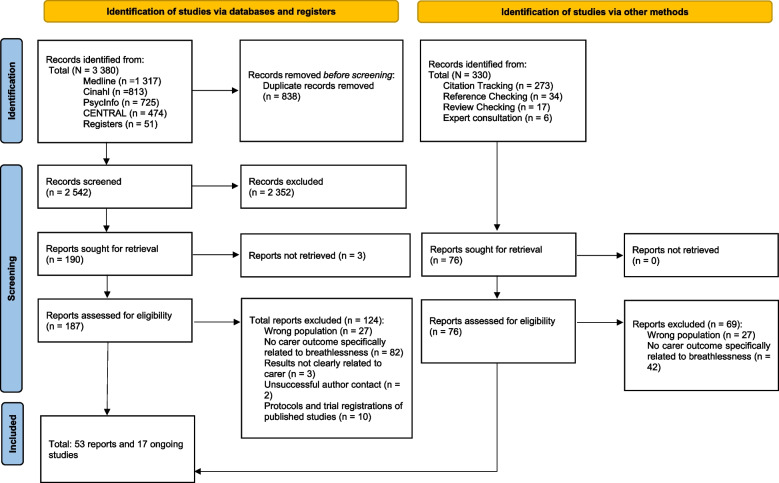
PRISMA Flowchart

Reasons for exclusion of full-text reports were documented (see Fig. 1 and Supplement II). We retained potentially relevant ongoing studies on the 'awaiting classification' list (see Supplement III).

These studies were collected but not included and reviewed again at a later date during search to determine whether new results were available and could possibly be included. We also included any published quantitative or qualitative primary study with original data and full-text reports. Additionally, studies were included in which a patient-oriented intervention was present that led to a subsequent effect on carers, meaning that carer-specific outcomes could be obtained.

### Assessment of methodological quality

The Mixed Methods Appraisal Tool (MMAT; Version 2018) by McGill University [23] was used to assess overall quality and the risk of bias of different types of studies (see Supplement IV). Two reviewers (SB and CW) independently performed the quality assessment. Disagreements between reviewers were resolved with a third reviewer (StS or AP). No study was excluded based on its quality.

### Data extraction and analysis

Two reviewers (SB and CW) extracted each half of the relevant data from the included studies and compared and discussed uncertainties. A second reviewer (SB or AP) checked at least 50% of the extracted data, and authors of the studies included were contacted to obtain missing data if necessary. A convergent segregated approach with narrative synthesis of the results was adopted based on the Johanna Briggs Institute (JBI) Framework for mixed-methods systematic reviews [21] and the work of Sandelowski et al. and Hong et al. [24, 25]. According to this approach, we first performed a segregated analysis and synthesis of the quantitative and qualitative data separately. For qualitative evidence, we used a pragmatic meta-aggregation approach that follows the JBI manual [21]. Two reviewers synthesised and categorised qualitative and quantitative data into broader categories (burden, needs and coping strategies) according to the type of measured outcomes or described theme in combination with breathlessness. No meta-analysis could be conducted due to study heterogeneity.for each data strand [21]. 

Second, we juxtaposed the quantitative and qualitative results synthesised in the first step. Thus, this employed a mixed-methods combination of qualitative and quantitative data to examine carers’ burden, unmet needs and coping strategies with a focus on the types associated with breathlessness We included all relevant findings in the analysis.

Thus, two reviewers first screened all findings and synthesised the findings by summarising and categorising them based on similarities in meaning, to create an aggregated summary of all included studies. Quantitative evidence was synthesized into a narrative summary based on used scales and outcomes according to carers.

In the mixed methods section, each outcome of the qualitative and quantitative data was categorised as matching or not matching to examine the agreement or disagreement between the two data strands [24]. Three subcategories were outlined for burden based on the caregiver burden inventory (CBI): emotional, social and physical burden [26]. Needs denote an imbalance between ‘what should be’ and ‘what is’ [27]. Coping describes the individual's way of dealing with a challenging situation by changing the situation itself or their attitude towards the situation. The synthesised results of coping are subdivided into problem-oriented and emotional-oriented coping according to the brief coping orientation to problems experienced (COPE) [28].

Interventional studies, independent of the type of study, were further categorised based on carers’ involvement in dyadic interventions.

## Results

### Description of studies

Our search until July 2023 retrieved 3,380 reports from databases and trial registers (see Fig. 1). Additionally, 330 reports were identified via forward and backward search. A total of 53 reports were included in the review. Seventeen ongoing studies were placed on the ‘awaiting classification’ list, none of them could be included during the project time (see Supplement III).

Of the 53 studies, we identified 20 qualitative studies (see Table 2) with a total of 217 carers, with samples ranging from four to 25 participants aged between 20 and 84 years, on average 59.5 years. The studies included one focus group [29] and 19 (two reports from one study) interview reports [14, 17, 29–46] as well as one published letter to the editor with qualitative interview data [47].

**Table 2:** Qualitative Studies

Author, year	Country	Aim	Data collection and analysis	Sample (n)	Patient diagnosis
**Aasbo et al. 2017** [30]	Norway	To investigate how carer negotiate their role as carer with patients and healthcare professionals when chronic illness turns into acute exacerbations for patients with COPD	Semi-structured interviews and thematic analysis	10 carers Age: Range 61 – 84y 4 males, 6 females	COPD with acute exacerbation and some under oxygen therapy
**Bailey 2004** [31]	Canada	To explore the affective component of breathlessness to patient-carer dyads	In-depth Interviews and narrative analysis with ethnography	10 carers Age: NA Gender: NA	COPD with breathlessness and acute exacerbation
**Bergs 2002** [32]	Iceland	To describe the experience of quality of life of women, taking care of husbands with chronic obstructive pulmonary disease (COPD)	Unstructured, in-depth interviews with carer and phenomenological method	6 carers Age: Range 47 – 69y 6 females	COPD
**Booth et al. 2003** [33]	England	To explore the experience of breathlessness and its effects on everyday life for patients and carers	Semi-structured Interviews and a coding framework was constructed	20 carers Age: NA Gender: NA	Cancer and COPD with breathlessness
**Booth et al. 2006** [34]	England	To evaluate the Breathlessness Intervention Service (BIS)	Unstructured interviews and open line-by-line coding	9 carers Age: NA Gender: NA	intractable breathlessness
**Clancy et al. 2009** [35]	UK	To explore longitudinally the views, feelings and experiences of people with COPD and their carers, at the onset of prescribed LTOT	focused-conversation style interviews and Heideggerian phenomenology	7 carers Age: Range 50 – 78y 5 females, 2 males	COPD with LTOT due to hypoxemia
**Collier et al. 2017** [36]	Australia	To understand carer experiences and perspectives with caring for individuals on long-term oxygen therapy (LTOT)	Semi-structured interviews and grounded theory	20 carers Age: NA 7 males, 13 females	Any life-limiting disease with breathlessness and receiving LTOT
**Ek et al. 2011** [37]	Sweden	To illuminate couples ‘experiences of living together when one partner has advanced COPD treated by means of long-term oxygen therapy	Repeated qualitative interviews and phenomenological-hermeneutical methods	4 carers Age: Range 67—74y 1 female, 3 males	COPD with LTOT
**Farquhar et al. 2017** [17]	UK	To identify the educational needs of carers of patients with breathlessness due to advanced disease to provide an evidence base for further interventional content	Separated in-depth interviews and framework analysis	25 carers Age: Range 42 – 84y 21 females, 4 males	COPD and cancer with breathlessness
**Ferreira et al. 2022** [38]	Australia	To explore patients and carers’ experiences with regular, low-dose, sustained-release morphine for severe chronic breathlessness associated with chronic obstructive pulmonary disease (COPD)	semi-structured interviews and constant comparative approach guided by grounded theory principles	9 carers Age: Median 70 (IQR 69—79) 3 males, 6 females	COPD with morphine for severe breathlessness
**Ferreira et al. 2020** [39]	Australia	To understand the experience of living with, and responding to, severe chronic breathlessness in people with COPD from the perspective of the patient and their carer	semi-structured interviews and constant comparative approach guided by grounded theory principles	9 carers Age: Median 70 (IQR 69—79) 3 males, 6 females	COPD with chronic breathlessness
**Ferreira F et al. 2020** [46]	Portugal	To explore the experiences of carers of people with breathlessness at home and identifying the strategies that these carers adopt to help controlling this symptom	Mixed questionnaire of open and closed-ended questions and qualitative content analysis	14 carers Age: aged over 63 years (42.8%) 57.1% female	cancer (breathlessness)
**Gysels et al. 2009** [40]	UK	To investigate the caring experience of carers for patients with an advanced progressive illness who suffer from breathlessness	In-depth semi-structured interviews and grounded theory	15 carers Age: Range 40—72y 15 females	Breathlessness due to COPD, cancer, MND or hearth failure
**Hynes et al. 2010** [41]	Ireland	To explore the experiences of carers providing care in the home to a family member with chronic obstructive pulmonary disease 1 Meaning and experience of carers´ role 2 Meeting the needs of care recipients 3 Interaction with formal health care	Semi-structured interviews and hermeneutic phenomenological	11 carers Age: Range 20—79y 9 females, 2 males	COPD
**Moody et al. 2004** [29]	USA	Identify perceived needs and what they would have liked the hospice nurses to help them with, what carer do to assist the patients, and what hospice staff could do better	Focus groups and data reduction by Krueger	6 carers Age: Range 38—65 y 5 females, 1 mal	Lung cancer, COPD with severe breathlessness
**Pooler et al. 2018** [42]	Canada	To explore bereaved carers’ experiences of IPF patients’ end-of-life care with the palliative approach initiated at the first visit to the clinic	Open-ended interviews and narrative approach with thematic content analysis	8 carers Age: between 50 and 80 s 7 female, 1 male	ILD
**Reitzel et al. 2022** [43]	Germany	To explore the experiences and perceptions of carers regarding episodic breathlessness and how they manage care of individuals with episodic breathlessness	Semi-structured interviews and content analysis	13 carers Age: Range 50—78y 7 females, 6 males	COPD, cancer, ILD, chronic heart failure with episodic breathlessness
**Rocker et al. 2012** [44]	Canada	To explore the experiences of patients and family carers with opioids for refractory COPD-related breathlessness and the perspectives and attitudes of physicians toward opioids in this context	Semi-structured interviews and interpretive description approach	12 carers Age: Range 34—75y 5 males, 7 females	Advanced COPD with opioids for refractory dyspnoea
**Schunk et al. 2019** [17]	Germany	To explore the experiences and needs of patients with breathlessness, their carers, and health care providers (HCPs) and their expectations for future service developments	Semi-structured interviews and qualitative content analysis	3 carers Age: median 53.6y 2 females, 1 male	COPD, Cancer and lung fibrosis with breathlessness
**Sigurgeirsdottir et al. 2020** [45]	Iceland	To explore principal family members’ experience of motivating patients with chronic obstructive pulmonary disease (COPD) towards self-management	In-depth interviews and phenomenological analysis	10 carers Age: NA 4 males, 6 females	COPD (70% with GOLD IV)

In addition, we identified 32 quantitative studies (see Table 3 and Supplement V) with a total sample of 4,632 carers and sample ranges from three up to 670 carers. The average carers’ age was 63.6 years, ranged from 28 up to 73.4 years.

**Table 3:** Outcomes of quantitative studies

**Outcome: Overall burden & breathlessness**			
**Studies**	**Measures**	**Populations**	**Results**
**Celik et al. 2022 ** [48]	ZBI ESAS	98 cancer carers	Breathlessness mean score were sig. higher in with higher carer burden (*p* < 0.05)
**Fernandez-Garcia et al. 2021 ** [49]	ZBI mMRC	91 COPD carers	Degree of breathlessness 3–4 [OR = 4.7 (95% CI = 1.7–13.2); *p* = 0.003] were independently related to carer burden 92.3% perceived an overburden
**Krug et al. 2016 ** [50]	BSFC QLQ-C15-PAL	58 carers of patients on palliative station Follow-up: 6 months	Influence on carer burden: Positive changes in carer burden (t1 and t3) and breathlessness (t1 and t2) imply an increase in carer burden and a higher severity of breathlessness (breathlessness (t2-t1) reg. coefficient: 0.05, 95% CL [0.01, 0.09]; p: 0.03)
**Malik et al. 2013 ** [13]	ZBI mBorg Scale	101 (50 lung cancer, 51 heart failure) carers	Mean burden scores were similar in both carer groups, and severe burden (score of > 16 on the ZBI-12) were reported from: - lung cancer carer 30% (95% CI = 17–43%) - heart failure carer 19% (95% CI = 8–30%) No association between burden and patients’ diagnosis or severity of breathlessness
**Manivannan et al. 2023 ** [51]	ZBI EORTEC QLQ C15PAL	2022 cancer carers	Correlations: Small sig. positive between EORTEC QLQ C15 PAL symptom score (breathlessness, insomnia etc.) and ZBI: Values between breathlessness and ZBI: Spermans correlation Rho 0.154 (95% Cl 0.018—0.284; *p* = 0.022)
**Tang et al. 2011 ** [52]	CBS MRCD	112 silicosis carers	Carer burden was significantly correlated with severity of breathlessness (r = .359, *p* < .0001)
**Takao et al. 2023 ** [53]	CCI Breathlessness: a scale ranging from 0 (not at all) to 4 (very acute)	670 bereaved family members: Subgroup of 86 carers of terminal cancer and dementia patients and 587 without perceived dementia	The carer burden was significantly higher (3.61 ± 1.58 vs 3.22 ± 1.47; *p* < 0.036) among family carers of terminal cancer patients with dementia Breathlessness (OR, 1.67, CI 1.10–2.55, p: 0.015) was contributing factor for carer burden when perceived dementia, and (OR 1.14, Cl 0.95–1.37, p: 0.153) for carer burden without perceived dementia
**Jesus et al. 2022 ** [54]	CBI mMRC	54 carers of patients with LTOT Carers ‘ quality of life: EQ-5D; European Quality of life index Breathlessness: mMRC	Univariate linear regression: total score of increased carer burden was correlated with higher breathlessness (*p* = 0.006) r. = 0.369 CBI dimension: Time-dependence burden: correlated with higher limitation due to breathlessness (*p* = 0.01) r. = 0.335 Development Burden: correlated with higher limitation due to breathlessness (*p* = 0.02) r. 0.309 Increased physical and emotional burden correlated with higher limitation due to breathlessness (*p* = 0.007 and 0.01) r. = 0.363 and r. = 0.333 Social burden: (*p* = 0.24) r. = 0.162, (not significant) Increased total score of carer burden was correlated with worse quality of life for carers
**Outcome: psychological distress & breathlessness**			
**Studies**	**Measures**	**Populations**	**Results**
**Al-Gamal et al. 2013 ** [55]	HADS D-12	67 COPD carers	Positive correlation (r = 0.307, *p* < 0.05, r = 0.286, *p* < 0.05)
**Bernabeu-Mora et al. 2016 ** [56]	Goldberg Test MRC	84 COPD carers 3 months: from hospitalisation due to exacerbation	Depressive symptoms: Baseline: 45 carers At 3 months: 32 carers Predictors of carers depression: • Spousal caregiving OR 2.65 (95% Cl 0.85–8.26; *p* < 0.10) • breathlessness OR 4.10 (95% CI 1.11–15.22; *p* < 0.05), • severe airflow limitation OR 3.88 (95% CI 1.42–10.65; *p* < 0.05) R^2^ = 24.10%
**Freeman et al. 2016 ** [57]	Depression rating scale and own classification	NA (6.655 patients and carers) from interRAI Palliative Care (PC) (2006 – 2011)	25.2% (n = 1.580) carers exhibit signs of distress, carers of breathlessness patients were more likely to it (*p* = 0.005) 24.6% (n = 716) from 1.580 of carers exhibit signs of distress when breathlessness in present while performing activities and 29.1% (n = 312) while breathlessness in present at rest Log. Regression: Significant relationship between care unit (patient and carer) distress was evident showing that persons with breathlessness were at increased risk for care unit distress (OR 1.18; 95% CI 1.07–1.30; *p* = 0.001) Breathlessness remained significantly associated with care unit distress (OR 1.18; 95% CI 1.06–1.32; *p* = 0.003) when controlling for client level characteristics including age, gender, diagnosis, and prognosis
**Granados-Santiago et al. 2023 ** [58]	HADS mBorg Scale	70 COPD carers Divided into two groups based on carers’ burden (35/35)	Anxiety and depression for carers HADS: 24.83 SD 10.11 vs. 15.6 SD 8.74
**Malik et al. 2013 ** [13]	HADS mBorg Scale	101 (50 lung cancer, 51 heart failure) carers	Anxiety: Lung cancer carers: mean 8.2 (SD 4.4) Heart failure carers: mean 7.7 (SD 4.1) Depression: Lung cancer carers: mean 5.1 (SD 3.6) Heart failure carers: mean 4.6 (SD 3.5) Overall anxiety score was higher in both groups compared to overall depression score Carers depression and looking after more breathless patients is associated with fewer positive caring experiences (R^2^ = 0.15; F = 4.4; *p* = 0.04)
**Mi et al. 2017 ** [59]	HADS CRQ	113 COPD carers	Prevalence of carer distress and anxiety (46% and 23%) No association between breathlessness and carers HADS values
**Mi et al. 2018 ** [60]	HADS MRC	117 COPD carers	Most bothersome symptom were breathlessness and fatigue
**Oechsle et al. 2013 ** [61]	PHQ-9, GAD-7 MSAS	33 cancer carers	Significant positive correlation between: Carers’ depression and total breathlessness (r = 0.37; *p* = 0.036)
**Seow et al. 2021** [62]	Distress: Yes/No Breathlessness: Yes/No	NA	Presence of breathlessness is associated with distress (OR = 1.19, 95% CL 1.18–1.2)
**Outcome: quality of life & breathlessness**			
**Studies**	**Measures**	**Populations**	**Results**
**Lyons et al. 2020 ** [63]	SF-36v2 SOBQ	109 lung cancer carers (after 12 month 68 carers) Over 12 months	Carers reported significantly poorer mental health when they were women, cared for younger patients, and cared for patients who were women (in relation to pain and breathlessness): χ2 (1, n = 109) = 10.65, *p* < .01) Greater incongruence in patient’s breathlessness and pain (between carer and patient rating) was significantly associated with worse physical health for carers
**Tang et al. 2011 ** [52]	MRCD SF-36	112 silicosis carers	Physical component summary score (PCS) (SF-36) PCS: Mean (SD) 54.4 (10.0) Poorer PCS was significantly correlated with patients’ severity of breathlessness (r = .223, *p* = 0.019), Mental component summary score (MCS) (SF-36) MCS: Mean (SD) 51.8 (10.1) Poorer MCS was significantly correlated with severity of breathlessness (r = .299, *p* = 0.001)
**Malik et al. 2013 ** [13]	SF-36 mBorg Scale	101 (50 lung cancer, 51 heart failure) carers	Quality of life: similar in both groups (lung cancer carers vs. heart failure carers)
**Moody et al. 2003 ** [29]	DGRIS The Hospice Quality of Life Index	163 lung cancer carers	Carers health related Quality of Life (Range 11 to 116): 55.06 (22.10) Factors influencing Carers' Quality of Life: symptom distress, age, educational level, and the patient's breathlessness intensity were significantly related to their perceived quality of life (R^2^ = .40, *p* = .02)
**Outcome: coping & breathlessness**			
**Studies**	**Measures**	**Populations**	**Results**
**Malik et al. 2013 ** [13]	Coping style: problem-focused, emotion-focused, dysfunctional-focused mBorg Scale	101 (50 Lung Cancer, 51 Heart Failure) carers	No difference between groups
**Outcome: use of supportive service & breathlessness**			
**Studies**	**Measures**	**Populations**	**Results**
**Yamamoto et al. 2021 ** [64]	*Satisfaction with care provided for terminal breathlessness*: Factor Score in Total: Agree 3; slightly agree, 2; and slightly disagree and disagree, 1 n = Sum of the responses to agree and slightly agree	231 dying cancer carers	Exploratory factor analyses (EFA) for Factor 3 was part of family care: Care for family members (Mean = 2.0, SD = 0.7) - Helping the family easily to understand the patient’s cause of breathlessness: Total n = 159 (68.8%) - Managing breathlessness while respecting families’ preferences: Total n = 150 (64.9%) - Promoting family involvement in the process of care: Total n = 141 (61%) - Listening to families’ anxiety and distress: Total n = 135 (58.4%)
**Yi et al. 2022 ** [65]	Discreet choice experiment: to elicit preferences and acceptability of breathlessness triggered services (BSs) Markov model Euro-Qol-5 and health and social care costs	68 carers of COPD, ILD, lung cancer	Carers´ preferences: Differs from patients’ preferences: X^2^ = 21.77; *p* < 0.04 Stated a strong preference for BS with home visits from GPs, and social worker and therapists’ involvement Markov model: cost-effectiveness for a 75-years old man over 5 years providing BS is cheaper (over 12 weeks) than usual care and quality of life improved
**Outcome: unmet needs & breathlessness**			
**Studies**	**Measures**	**Populations**	**Results**
**Mi et al. 2018 ** [60]	CSNAT MRC	117 COPD carers	In multivariate analysis the association between unmet support needs and greater estimation of breathlessness by carers remained when adjusted for patient and carer age and sex (odds ratio 1.250, 95% CI 1.031–1.516), as did younger patient age and greater patient estimation of depression (OR 1.09, 95% CI 1.018–1.167)

*SOBQ* Adapted version of the USCD Shortness of Breath Questionnaire

*SF-36v2* Short Form Health Survey 2

*DGRIS* 11-point Dyspnea Graphic Rating Intensity Scale

*CSNAT* Carer Support Needs Assessment Tool

*ZBI* Zarit Burden Interview

*MSAS* Memorial Symptom Assessment Scale

*HADS* Hospital Anxiety and Depression Scale

*D-12* Dyspnoea-12

*mMRC/MRC/MRCD (modified)* Medical Research Council Dyspnea

*CRQ* Chronic Respiratory Distress Questionnaire

*mBorg* Scale modified Borg Breathlessness Scale

*PHQ-9* Patient Health Questionnaire-9 (German Version)

*GAD-7* Generalized Anxiety Disorder Scale-7 (German Version)

*ESAS* Edmonton Symptom Assessment System

*CBS* Caregiver Burden Scale

*BSFC* Short form of the Burden Scale for Family Caregivers

*QLQ-C15-PAL* EORTC quality of life Questionnaire Core 15 Palliative

*CCI* Caregiver Consequence Inventory

*CBI* Caregiver Burden Inventory

We included 17 cross-sectional reports [13, 48, 49, 51–55, 57–61, 64, 66–68], two publications from one study population [59, 60], five mixed-method studies [69–73], with two publication from one study, three randomised (controlled) trials [74–76], one prospective study [56], two retrospective studies [62, 77], one economic study [65], three secondary analyses [50, 63, 78] based on two prospective observational cohort studies [50, 63] and one randomised controlled trial with data from the intervention arm [78] (see Table 4: RCTs and mixed-method studies).

**Table 4:** RCTs and mixed-method studies

Study, year	Type of study	Patients´ diagnosis	Carer´ characteristics	Intervention		Control	Outcomes and measures	Results
**Randomised Studies**								
** Choratas et al. 2020** [74] **(Cyprus)**	Randomised feasibility study	Cancer	N = 11 in the intervention group N = 8 in the control group Age: 74% (n = 14) over the age of 61 Gender: 63% (n = 12) female	*Educational program:* PowerPoint presentation with two video recordings and a practical exercise for patients and family caregivers. Practical parts were diaphragmatic breathing, inspirational muscle training (IMT), and use of a handheld fan		*Usual care:* pharmacological management by oncologists	Time points of measurements: Baseline (before Intervention), in 2 weeks, and in 4 weeks *Carer:* assessed patient’s breathlessness using the mBorg scale and the effect of the educational programme by using the HADS scale for anxiety and depression and the Zarit Burden Interview (ZBI) scale for the burden they experienced	**Baseline:** *mBorg scale (Range: 2–9)*: median of 4.8 (± 1.8) *HADS scale (Range: 0–21):* - Anxiety: 7.9 (± 4.3) - Depression 7.2 (± 4.4) *ZBI Scale (Range: 0–88):*31.7 (± 11.9) **Effect of the Intervention (after 4 weeks)** *Patient´s breathlessness (mBorg Scale):* - IG (− 0.6) improvement - CG (+ 1.5) deterioration *Anxiety and Depression (HADS)*: - IG (− 0,4/ + 0,1) steady - CG (+ 3,5/ + 2,3) deterioration Burden (ZBI): - IG (− 2,3) improvement - CG (+ 10,8) deterioration
** Given et al. 2006 ** [75] **(USA)**	RCT	Cancer	N: IG 59/CG 66 Age: mean IG: 55.3 (SD 13.76) CG: 54.4 (SD 13.13) Gender:44.1% male	*Cognitive behavioural intervention (CBI):* strategies for managing symptoms, how to integrate assistance into daily lives, and better communicate with their patient and health care providers regarding symptom management		*Standard care* (no further information’s)	Time points of measurements: at baseline, after 10 and 20 weeks (Data were analysed for baseline and 10 weeks) Depressive symptoms: Depression scale (CES-D) Caregivers’ reaction to assisting with symptom management: measured by (1) total number of common symptoms for which carer provided assistance;(2) total level of distress; (3) reaction score per symptom	Results are extracted only for breathlessness: Total Number (n) of symptom assistance: - at baseline: 65 CG - 10 Week: 61 CG Caregivers assisting with symptom (n): - Baseline: 30 CG - 10 Week 10: 16 CG Caregiver’s Negative Reaction Score (10-point scale: 0 = no distress to 10 = worst distress): - Baseline: 3,10 - 10 Week: 3,19 Overall: female caregivers were more responsive to the intervention
**Schunk et al. 2021 ** [76] **(Germany)**	RCT	Any advanced life-limiting disease	N: 95 (IG: 44, CG: 51) Age: Gender:	*Munich Breathlessness Service (MBS)* short-term intervention, 2 personal contacts with palliative care specialists for exercise and positions to facilitate breathing; breathing techniques; exercise plan; assessment of need for medical aids , 3–4 specialist respiratory physiotherapy, 2 Letters to patients, treatments within 6 weeks) + Standard Care: respiratory specialists, general practitioners, any disease- oriented treatment and palliative care services		Control group: gained access after a waiting time of 8 weeks + standard care	Carers completed paper-based Questionnaires: Change in carer burden assessed with the Zarit Burden Inventory (ZBI)	**T0:** ZBI Sum score: mean 21.35 (SD 12.86) ZBI sum score could only identify small effects of the intervention that were not statistically significant
**Mixed-Method Studies**								
** Farquhar et al. 2016 ** [69] **(UK)**	Mixed method RCT	Advanced non-malignant disease	N: 45 – 57 respondents Total Age: 62.2 (13.39) Female: 79% (45)	*The Breathlessness Intervention Service (BIS):* Multi-disciplinary, complex intervention that is supported by a palliative care approach in theory and uses evidence-based (non-) pharmacologic interventions to help patients with advanced disease manage their respiratory distress		*Standard care:* specialist outpatient appointments in secondary care	Carer distress: NRS Carer Anxiety: HADS Brief qualitative topic-guided interviews with all patients and carers to explore their expectations and experiences of BIS	**8 Weeks from baseline** Carer NRS distress due to patient´s breathlessness (0–10): - IG: reduction (1.03-point) - CG: 0.2-point increase This was not statistically significant: adjusted difference of –0.42 (95% CI: –1.86 to 1.02), *p* = 0.56 Carer HADS-Anxiety (0–21): IG: 1.65-point, reduction CG: 0.15-point reduction This was not statistically significant: adjusted difference of –1.22 (95% CI: –2.84 to 0.40), *p* = 0.14 Qualitative interviews: described a sense of relief from talking to someone about breathlessness during the intervention
** Farquhar et al. 2014 ** [70] **(UK)**	Mixed-method RCT (Phase III)	Cancer	N: 39 – 41 respondents Age: 64.6 (12.7) Gender: 68% (28) female	*The Breathlessness Intervention Service (BIS):* multi-disciplinary complex intervention combining (non-) pharmacological interventions to support breathless patients with advanced disease, theoretically grounded by a palliative care approach		*Standard care:* specialist outpatient appointments in secondary care	Primary outcome: change in distress due to breathlessness (NRS range 0 to 10) and HADS Qualitative interviews with patients and carers	Primary outcome: There was little change in carer distress. (No given data) Qualitative interviews: Carer described not feeling alone by having the ability to call someone in the BIS model
** Schloesser et al. 2022 ** [71] **(Germany)**	Mixed Methods single arm phase II study	episodic breathlessness due to any life-limiting and progressive disease	N: 16 (were named by patients) Age: mean age 63.5; SD = 8.7 Gender: female 9 (56.3%) male 7 (43.8%)	*Brief Cognitive and Behavioural Intervention:* - 1-to-2-h intervention - Delivered by a nurse, psychologist, or a physician - Consists of a general introduction to better understand breathlessness, as well as strategies and education		NA	**Outcomes: via closed-ended questions** Safety and Acceptability: participants were asked about burdens due to the intervention and study procedure Qualitative Interviews: to evaluate participants experiences	Outcomes were evaluated six weeks after intervention and as treated Carer reported Outcomes: - No carer reported any burdens due to the intervention/study procedure. The great majority of the carers were very satisfied with the intervention and the study procedure (≥ 8/10) - Safety from carers ´perspective: no adverse effects from intervention for IC, no unexpected side effects from intervention for informal carer and no adverse effects from research for informal carer Qualitative interviews were conducted only with patients
** Swan et al. 2019 ** [72] **(UK)**	Mixed-method RCT	Adult respiratory outpatients with Medical Research Council breathlessness scale grade ≥ 3	14 carers were recruited and 13 (92%) completed the study	*4 groups:* Fan = battery-operated hand-held fan Calming Hand (CH) = a cognitive strategy *(1) exercise advice only* *(2) CH & exercise advice* *(3) Fan and exercise advice* *(4) fan, CH and exercise advice* All four groups received one-hour face-to-face individual training in standardised breathlessness self-management and exercise advice. All participants were given an information leaflet for use at home CH and fan groups: received instructions how to use the interventions			Carers self-efficacy: General Self-Efficacy Scale (GSES) Carer Assessment: Zarit burden short-form	Caregiver Outcome: Zarit burden and GSES: Improvements in carer´ outcomes were in the fan & CH & exercise advice arm only mean change absolute from baseline to day 28 - Zarit burden 0.25; (7.1%) - GSES 1.75; (5%)
** Hutchinson 2022 ** [73] **(UK)**	Mixed-method feasibility cluster randomised controlled trial	Acute-on-chronic breathlessness due to COPD or heart failure	N: 9 caregivers Age: 28 – 67 Gender: only females	BREATHE: reassure patient and carer, check posture, exercises, airflow, technics to manage panic and fear, education of patient and carer (information booklet)	Usual care: immediate clinical assessment, reassurance, oxygen, nebulizer		Acceptability: Fidelity: Completion rate Safety: any adverse events	Acceptability: Qualitative and survey data showed acceptability to patients, carers and paramedics One carer read booklet and leaflet and derived benefits. One carer read only the leaflet and dealt with two further episodes without calling the ambulance Safety: No adverse events Fidelity: NA for carer

The most common underlying disease of the breathless patients was COPD (26 studies) followed by cancer (18 studies). Others were interstitial lung diseases, heart failure, amyotrophic lateral sclerosis, motor neurone diseases and silicosis. The majority of carers were female**,** only two studies reported more males [37, 55]. Studies were from 20 different countries, most from UK (n = 8), USA (n = 6), Canada (n = 5), Germany (n = 5), Australia (n = 3). The overall quality score (see Supplement IV) e.g. the mean score was 72% (± 20%) over all reports.

### Findings of included studies

**Fig. 2:**
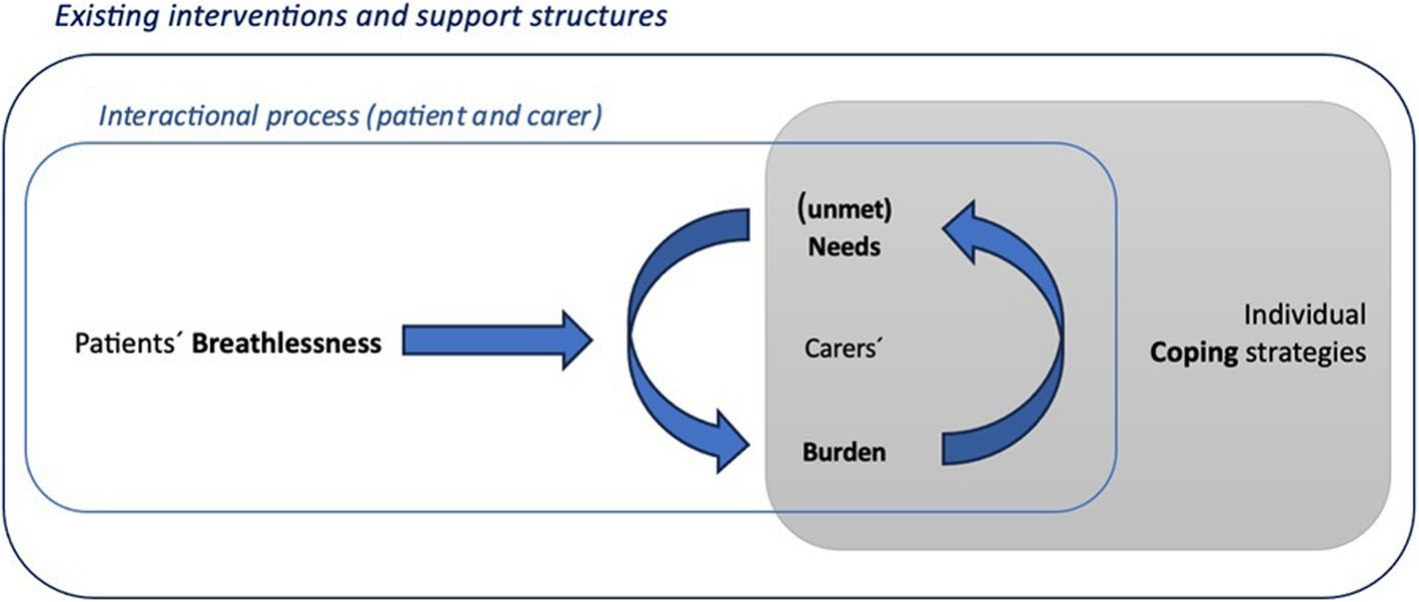
Caregiving process

**Fig. 3:**
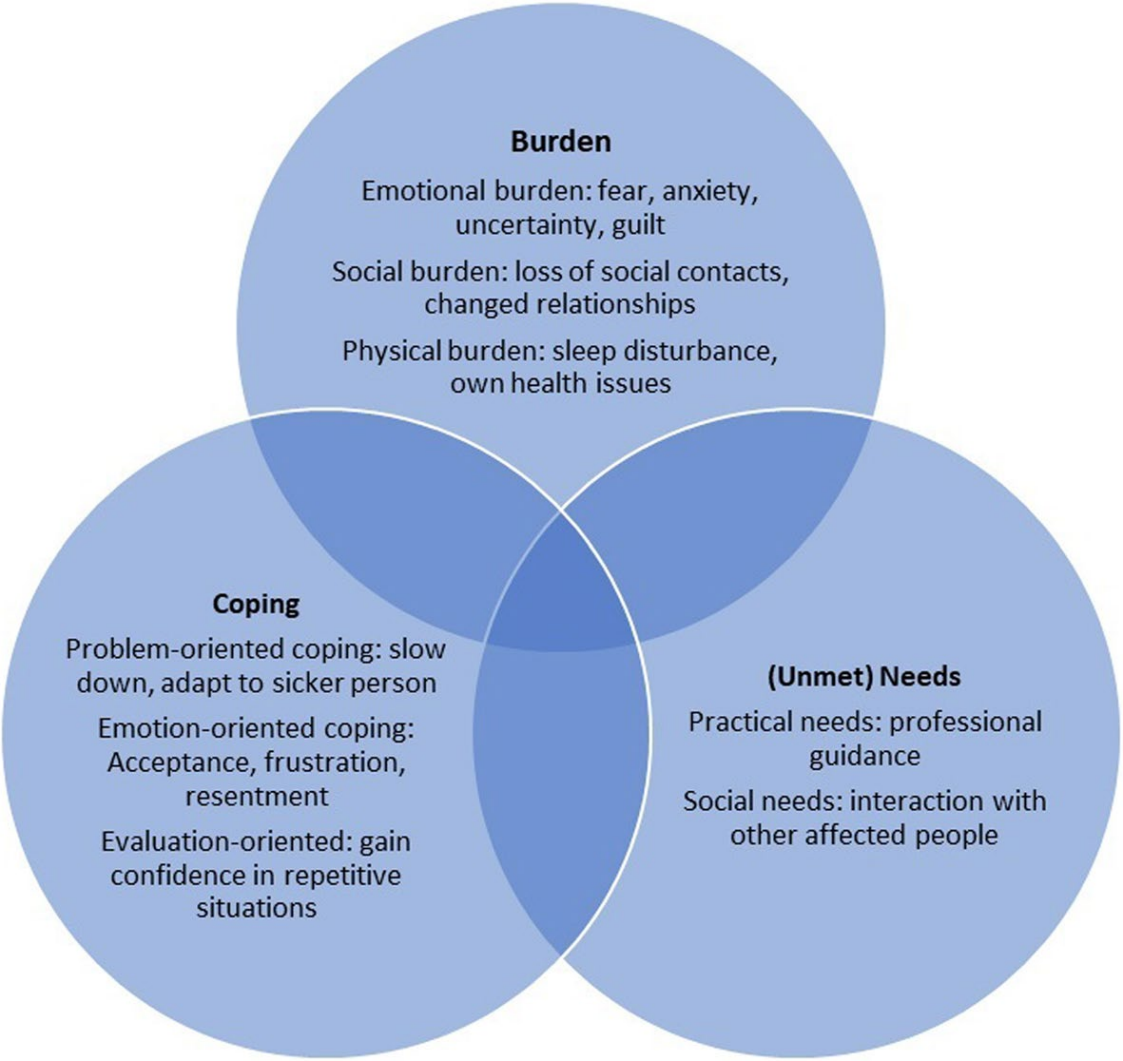
Overview of qualitative and quantitative findings

To answer our review question, we structured the results using a theoretical framework that we developed for this purpose (see Fig. 2: Caregiving process), with four main categories: burden, (unmet) needs, coping strategies and the use of healthcare and social services. For each category, we first reported qualitative studies, then the quantitative ones, and finally an integration of both types of studies. A synthetic overview of the key findings is presented in Fig. 3.

### Burden

Qualitative studies have reported an emotional burden on carers. Frequently mentioned were feelings of insecurity and fear related to the patient’s breathlessness as well as the carers´ uncertain future. A general uncertainty was a prominent theme in all studies. The sudden appearance of breathlessness and its unpredictability were both the cause and effect of a permanent state of uncertainty and alertness for carers [17, 29–33, 35, 37, 39, 40, 45, 46, 67]. Carers also suffered from severe sleep disorders and constant fear of their patient’s sudden death due to breathlessness, especially at night [30, 32, 33]. Inherent uncertainty due to anxiety and fear enormously increased the burden on carers.

I’d like somebody to tell me when it’s gonna happen. It’s like living with a pressure cooker or a time bomb. […] I know what his problems are. […] Although Mr. Johns told us it wouldn’t improve his breathing and everything like that, the build-up towards the operation was horrendous. (Carer of a patient with heart failure; Gysels et al. 2009, P. 155)

They were in a constant state of alertness and described patient’s breathlessness as a ‘frightening, little known, and poorly understood symptom’ [34] causing ‘primal distress’ [36]. Carers felt helpless and panicked because of a lack of knowledge about breathlessness and professional support structures [29, 31–33, 35, 36, 38–41]. As the following quotation highlights:

I thought he’d gone … it was terrifying really, it really is … you just don’t know what to do, you can’t do anything any way … you’re just helpless aren’t you. (Wife of emphysema patient aged 77, Interview; Booth et al. 2003, P. 341)

In addition, participants reported negative emotions owing to a lack of treatment interventions for their patients. Carers also reported a strong fear of being the trigger for patients´ next episode of breathlessness, e.g. through arguments, which may upset the patient and consequently cause breathlessness. This might result in emotional withdrawal and feelings of guilt among the carers [29, 32, 39, 44]. Therapeutic options such as oxygen therapy were identified as life-saving, but the carers felt burdened by the responsibility of determining the correct dosage for breathlessness relief and the underlying disease [36]. Nevertheless, when patients took opioids to reduce breathlessness, carers felt less stressed and noted an improvement in their own quality of life [44].

Four qualitative studies demonstrated how the relationship between carers and patients had evolved from a loving partnership to a new type of relationship with a new form of intimacy in the form of physical care and mutual dependence [34, 37, 39, 41].

I do not love him as I did earlier … I would rather say that I care immensely about him. This is more like fondness and friendship. (Participant 2; Bergs et al. 2002, P. 617)

Carers’ social lives were also more limited because of patients’ symptom deterioration [34]. Breathlessness was stated as the leading symptom among all diseases to a ‘shrinking world’ [32] and a major threat to care that intensified other symptoms and was the worst to handle [40]. Physical care, sleep disturbances, and carers’ health problems were further burdens. Additional challenges for carers due to the patient's underlying illness and breathlessness were limitations such as lack of barrier-free access to buildings to minimize patients´ physical burden, and the lack of visibility of patients´ underlying disease turned out to be a challenge in the consideration by others [17, 31, 35, 37]. Breathlessness required carer´s constant presence and created a strong mutual dependence and mutual suffering [41]. Breathlessness triggered dyadic anxiety or both patient and carers’ anxiety [17]. The following quotation describes this:

The first few times I called an ambulance, it was simply panic … although he was gasping for breath…he calms down the minute I phone an ambulance … It’s visible, because he knows and I know help’s on its way. (Carer 221; COPD; Farquhar et al. 2017, P. 7)

Carers also perceived a lack of problem awareness among healthcare professionals. They mentioned receiving little therapeutic advice to cope with breathlessness and having no access to support services (e.g., nursing) [17].

Seven quantitative cross-sectional studies [13, 48, 49, 51–54] and one prospective cohort study [50] investigated the association between overall carers’ burden, as measured by validated measurement tools (Zarit Burden Interview, Caregiver Burden Scale, Short Form of the Burden Scale for Family Caregivers, Caregivers Consequence Inventory, Caregiver Burden Inventory (CBI)), and patients’ breathlessness (see Table 3).

A significant association between carers’ overall burden and breathlessness was recorded in five [48, 50–52, 54] out of seven studies, and one study out of the seven studies identified breathlessness as an independent variable related to carers’ burden (OR = 4.7 (95% CI = 1.7–13.2; *p* = 0.003) [49]. One [54] out of the seven studies considered carers’ burden based on the CBI. Increased physical and emotional burdens were correlated with higher breathlessness (r. 0.363 with *p* = 0.007; r. 0.333 with p. = 0.01).

Another study [13] focused on the differences in overall carers’ burden based on patients’ underlying conditions by comparing heart failure patients and lung cancer patients with breathlessness, but found no significant differences in overall carers’ burden by diseases. Nevertheless, the studies collectively provide evidence of the impact of breathlessness on the overall burden of carers, regardless of the patients´ underlying diseases and the severity of breathlessness.

We found eight studies measuring carers’ psychological distress using validated measurement tools (Memorial Symptom Assessment Scale, Hospital Anxiety and Depression Scale, Patient Health Questionnaire-9 [German Version], Generalised Anxiety Disorder Scale-7 [German Version], Goldberg Test) in relation to patients’ breathlessness, including six cross-sectional [13, 55, 57, 59–61], one prospective [56] and one retrospective [62] studies (see Table 3). Six of the eight studies traced a significant association between breathlessness and psychological distress [13, 55, 57, 60–62], and one determined that breathlessness, severe airflow limitation, and spousal caregiving were the strongest independent predictors of carers’ depression [56]. Patients with breathlessness were at a higher risk of dyadic distress (OR 1.18; 95% CI 1.07–1.30; *p* = 001) [56]. Only one study found no association between psychological distress and breathlessness [59]. Overall, these studies provide evidence for the impact of breathlessness on carers' psychological distress. Higher levels of breathlessness are associated with increased psychological distress, including anxiety and depression, in carers [13]. Greater breathlessness and higher depression scores were associated with fewer positive caring experiences (R^2^ = 0.15, F = 4.4, *p* = 0.04). Male carers showed higher levels of anxiety (55% of 11 male carers versus 36% of 22 female carers) and depression (36% of male carers versus 14% of female carers) than female carers [61]. One in four carers showed signs of distress; carers of patients with breathlessness were particularly affected, with approximately 10% of carers reporting feeling unable to continue their care work [57]. Breathlessness was revealed to be a strong predictor of carers’ depression [56].

Three cross-sectional studies [13, 52, 67] and a secondary analysis of a longitudinal study [63] measured the association between quality of life and breathlessness (see Table 3). Carers who were women and cared for younger female patients reported worsened mental health [63]. Carers had significantly lower mental health than patients [63]. Two studies identified a significant positive correlation between carers’ quality of life and severe breathlessness [52, 67] (see Table 3). Malik et al. (2013) found no group differences in the quality of life between carers of patients with lung cancer versus with heart failure [13].

*Integration of qualitative and quantitative burden data:* The analysis indicated that both qualitative and quantitative studies consistently identified anxiety and depression as key aspects of the emotional burden experienced by carers of patients with breathlessness. Carers endured a significant emotional burden characterized by insecurity, fear, and constant vigilance due to the unpredictable nature of breathlessness. This persistent uncertainty leads to severe distress, including sleep disorders, and a persistent fear of sudden death, particularly at night. This emotional burden is further compounded by a lack of carers’ knowledge and support from healthcare professionals, resulting in feelings of helplessness and panic.

#### Unmet needs

Qualitative studies stated that carers highlighted practical needs, such as the need for someone to call for guidance and more self-management strategies for anxiety and distress [17, 29, 34]. They were eager for someone to listen to their situation [34]. Carers wished for more therapeutic involvement and knowledge to gain confidence in breathless situations [14].

I’d like to know a little more … when should I really be stopping him from doing something? […] I’ve had to say to him before ‘I’m not being unkind, but if you push yourself, you really make yourself unwell’, which he has done. I am the one that looks after him, and then that means I can’t get on with things because he is being silly. (Carer 222; COPD; Farquhar et al. 2017, P. 8)

They also desired professional support and guidance in understanding breathlessness, managing anxiety and panic, keeping patients active, and knowing what to expect in the future [14, 45]. Carers’ social needs with other affected people were also noted [17, 29]. Carers reported a strong desire for affirmation and appreciation in their breathlessness management work [14]. Regarding opioid medication for breathlessness, carers wished for more information about side effects and more openness from healthcare professionals towards the use of opioids [44]. Carers of patients with cancer were particularly eager for education and information to support patients, and COPD carers underlined a wish to better cope with their own anxiety regarding breathlessness [14].

We found only one cross-sectional study [60] reporting carers' needs using the Carer Support Needs Assessment Tool. Carers of patients with advanced COPD identified unmet needs in relation to their own physical health, managing their feelings and worries, and being able to take a break from caring overnight [60]. A significant positive relationship was found between unmet needs and higher breathlessness, which remained significant even after adjusting for age and gender [60].

*Integration of qualitative and quantitative unmet needs data:* Both qualitative and quantitative studies highlight that carers often face significant unmet needs, particularly in terms of practical support, managing their own emotional and physical health, and accessing sufficient information and guidance from healthcare professionals. However, the limited evidence in both data strands indicates that these unmet needs are likely to be underreported and insufficiently considered.

#### Coping

To deal in a problem-oriented way with breathlessness, carers reported the use of supportive interventions such as a 24-h telephone service and the general structures of inpatient palliative care as well as home-based specialised palliative care as helpful. Seeking and gaining better information about patients’ breathlessness care in pharmacological and non-pharmacological terms provided relief for carers [17, 34, 43]. Monitoring and regulation of medications, removal of breathlessness triggers, and breathing techniques have helped carers to manage breathlessness situations [29, 42]. Carers felt that their self-management improved by seeking social support, using psychosocial interventions, and sharing responsibilities for dealing with patients' breathlessness [43]. Mutual adaptation was a reciprocal coping process for challenges raised by breathlessness [32]. Thus, carers adapted their general rhythm to the affected person and slowed down to avoid triggering breathlessness in patients. Through repetitive incidents of breathlessness, carers gained confidence and learned when it is time to call for help [30, 34, 38].

In terms of emotional coping strategies, carers attempted to continue to bring joy and happiness into patients’ lives [39], focus on available resources to reduce their own anxiety [29, 34], stay emotionally calm in breathlessness situations [43], and calm patients by sitting down together [46]. The carers also described it as helpful to observe and experience other affected families and neighbours, which helped offer insights into their own future in their caring role [35]. In contrast, resignation emerged as a coping reaction in the context of unchangeable breathlessness. Carers had to give up their own identities to fulfil their new roles. In particular, female spousal carers suppressed their own emotions and feelings to avoid triggering their husbands´ breathlessness [32, 39].

A used individual coping strategy was ‘acceptance’—reported by 90% of the 101 caregivers in Malik et al. [13], which was also stated in Gysels et al. [36]. This suggests that carers, regardless of a patient’s specific disease, tend to adopt a similar coping approach when dealing with breathlessness and showed some kind of resignation to the breathlessness situation, as described in the following quote:

*Just watching him trying to get his breath … That is horrible to watch. And there is nothing you can do to help. Just keep him comfortable and be there, whatever he needs … I try not to worry too much; I try to keep calm because I don’t want him to get worse. (Carer 2; Ferreira et al., 2020, P. 7)*

One quantitative cross-sectional survey investigated family reported use of care strategies to relieve terminal breathlessness and families’ satisfaction with the care provided in intensive care units for cancer patients with terminal breathlessness. On average, all carers (n = 231) reported a slight level of agreement with receiving care support for themselves from healthcare professionals; this included helping carers to understand the reasons for breathlessness as well as listening to carers about their own anxiety and distress [64]. An economic evaluation stated a strong preference for home visits from general practitioners, as well as social worker and therapist involvement from Breathlessness Services [65]. Overall, the studies indicate that carers’ coping is highly individualised, and only a few behavioural patterns can be generalised.

*Integration of qualitative and quantitative coping data:* The integration of qualitative and quantitative data on coping strategies among carers of patients with breathlessness revealed limited evidence. Qualitative studies showed that carers used various strategies, including supportive services, better information on breathlessness management, and social support. They adapted their lives to the patients' needs, gained confidence through experience, and employed emotional coping strategies such as staying calm and accepting the situation, sometimes leading to resignation. Quantitative data were sparse and focused on general coping mechanisms and healthcare support. Overall, while carers developed individual strategies, there was limited evidence on coping, especially in quantitative studies.

#### Support services

Three RCTs [74–76] and three mixed-method RCTs [69, 70, 72], with two publications based on a single study and one single-arm mixed-method study [71], examined various interventions and general support structures.

Interventional components were either related purely to the training of patients and relatives (e.g., educational programmes or cognitive and behavioural interventions) [71, 74, 75] or were based on the care structure of the hospital in the form of a multidisciplinary intervention with individual components (e.g., breathlessness intervention service or Munich breathlessness service, see Table 4) [69, 70, 76]. One study focused on the effectiveness of individual intervention components (fan, exercise and calming hand as a cognitive strategy) and their combination in four different study arms [72]. In other trials, the comparison group received standard care. The main result was reduction in carers’ burden, measured with ZBI, HADS or depression scale.

Interventional studies [69–72, 74–78] in this review focused on the effectiveness of educational programmes, complex interventions and behavioural interventions compared with existing usual care in managing patients’ breathlessness. The interventions included educational materials, practical exercises and access to specialised clinics or services, fans and exercises, and behavioural parts in managing breathlessness situations. The results suggest that these interventions can provide benefits in terms of symptom management, self-care, coping strategies, and communication among carers, patients, and professionals. The other interventional focus was the implementation of service structures, such as access to specialised clinics or services, such as the multi-professional outpatient clinic (MBS) as reported by Schunk et al. (2021) or the Breathlessness Intervention Service (BIS) by Farquhar et al. (2014 and 2016), offered comprehensive care from various healthcare professionals, including palliative medicine specialists, physiotherapists, respiratory specialists, psychologists, and social workers [69, 70, 76]. These interventions combined non-pharmacological and pharmacological approaches to address symptoms and provided support to patients with advanced diseases. Carers received education and training on symptom management and integrating assistance into daily life, which emphasised the importance of carers’ involvement and support [77].

A within-subject analysis of the intervention arm of one RCT focused on telephone symptom management (TSM) for patients with lung cancer and their carers [78]. The results demonstrated that cognitive-behavioural interventions featuring more guided imagery (e.g., the use of summarised handouts) were associated with less psychological distress in carers, while greater practice of problem-solving strategies was associated with higher carers’ distress related to breathlessness [78].

Smallwood et al. (2019) undertook a survey assessing patients' and carers' experiences of the Advanced Lung Disease Service (ALDS), a model of integrated respiratory and palliative care [68]. The ALDS provided symptom management, education, and advance care planning. All carers reported that the ALDS helped them with symptom support in case of breathlessness; 66.7% of carers did not request additional information on managing breathlessness. Important aspects of care according to the carers of patients with breathlessness were continuity of care (95.8%) and long-term care (87.5%). The majority of carers did not need to see additional healthcare professionals during ALDS visits.

One letter to the editor described the feasibility of the hospice case manager training carers to use a breathlessness scale, upright positioning, and a fan for patients dying with COPD, lung cancer and/or heart failure. The carers (n = 7) stated that the intervention increased their confidence [47].

To better understand the interventional aspects of carers, three groups of carers’ involvement in dyadic interventions were distinguished to gain an overview of the interventional aspects of carers (see Table 5).

**Table 5:** Carers´ involvement in dyadic interventions

Authors/Studies	Intervention for	Breathlessness-specific intervention	Intervention helpful for carers	Carers’ involvement
**Choratas et al. (2020) ** [74]	Patients and carers	Yes	Yes	Supportive
**Farquhar et al. (2014) ** [69]	Patients and carers	Yes	Not significant	Supportive
**Farquhar et al. (2016) ** [70]	Patients and carers	Yes	Not significant	Supportive
**Given et al. (2006) ** [75]	Patients and carers	No^a^	No	Supportive
**Schloesser et al. (2022) ** [71]	Patients and carers	Yes	Partially yes	Supportive
**Schunk et al. (2021) ** [76]	Patients and carers	Yes	Not significant	Passive
**Smallwood et al. (2019) ** [68]	Patients and carers	Yes	Yes	Almost Active
**Swan et al. (2019) ** [72]	Patients and carers	Yes	Yes	Supportive
**Winger et al. (2018) ** [78]	Patients and carers	Partially Yes^b^	Partially yes	Almost Active
**Grosbois et al. 2022 ** [77]	Patients and carers	Yes	Yes	Active

Carers’ involvement:

Passive: In a passive carer role, carers are only passively involved in the intervention. They do not receive any specific interventional parts for themselves. The focus is primarily on evaluating carer´ outcomes related to the care provided to the patient

Supportive: In a supportive carer role, carers are involved in the intervention, but the interventions provided are primarily aimed at supporting the patient's condition. There are no interventional parts specifically designed for the carer themselves. However, through the improved care provided to the patient, carers may experience secondary benefits such as reduced burden or improved caregiving skills

Active: In an active carer role, carers are actively involved in the intervention and receive specific interventions for themselves that primarily benefit them. The interventions may be aimed at addressing carer-specific needs, such as psychological support, education, training or respite care. The focus is on improving the well-being and functioning of both the patient and the carer

^a^Breathlessness-specific results

^b^Some parts are breathlessness-specific

We found that it is common in interventional studies to primarily focus on outcomes and benefits for patients while only acknowledging the supportive role of carers. In such studies, interventions are primarily designed to improve patient outcomes; however, they may also indirectly have positive effects on carers. These include reducing carers’ burden, improving their skills and knowledge in providing care, enhancing communication and collaboration between carers and healthcare professionals, and promoting better overall well-being for both patients and carers. Table 5 presents an overview of supportive care interventions for both patients and carers. Four out of ten studies focused on interventions that were helpful to carers. Based on our grouping of the interventional components, we identified that carers primarily serve supportive functions aimed at aiding patients’ breathlessness. There were no aspects of a given intervention specifically designed for carers themselves. Nevertheless, the improved care provided to patients results in secondary benefits for carers, such as reduced burden and enhanced caregiving skills.

## Discussion

To our knowledge, this is the first systematic review aiming to synthesise all qualitative and quantitative literature on the carers of patients with breathlessness. First, an unsurprising key finding, with strong evidence in the literature, is that breathlessness has a significant impact on carers. They experience immense stress and burden, which not only affect their own well-being but also their ability to cope with repetitive and stressful situations. Second, carers experience poorer mental health and overall quality of life because of caring for someone with breathlessness. This aligns with the general literature on carers [79–82]. Third, carers of patients experiencing breathlessness rely on individual coping strategies, such as acceptance, and sometimes leading to resignation, believing that there is nothing they can do to alter the situation. Fourth, it is vital to provide supportive services to carers of patients experiencing breathlessness. We found positive effects on the carers of dyadic services on patients and carers. The results of our analysis revealed a lack of systematic and explicit services only for carers, particularly regarding their own burden and needs beyond the mere fulfilment of their role as supportive, caring, and present relatives or friends for the patients.

Anxiety and uncertainty are prevalent symptoms among carers [59]. They often find themselves in a constant state of vigilance and alertness, experiencing a continuous cycle of providing care [17, 29, 41]. Due to the constant monitoring by carers, sleep problems are an additional burden [32]. This is problematic as lasting fear and anxiety lead to higher risk of cardiovascular mortality, coronary heart disease, stroke and heart failure [83], which causes additional healthcare costs. Thus, early carers’ interventions should be provided.

Carers’ burden has been found across various diseases (COPD, chronic heart failure, cancer) in numerous studies, and breathlessness has emerged as a common symptom among these diseases. But breathlessness is not solely a symptom rather a complex phenomenon associated with multifaceted triggers and impacts for patients and carers, framed as ‘chronic breathlessness syndrome’ [3]. This is also stated in the qualitative synthesis [84] based on breathlessness as a ‘total dyspnoea’ concept [85] and in the extended version of ‘total breathlessness’ by Lovell et al. [86]. Breathlessness encompasses psychological, social, and physical components of patients [84] and carers. As such, a holistic and broad understanding of breathlessness helps in the assessment and management of carers: first, to detect the triggers (usually more than one) which cause or worsen the symptoms; second, to provide different ways and techniques to relieve the burden and enhance coping with breathlessness.

Regarding the urgent need for support structures for carers, education, information, and involvement in discussions about the causes of breathlessness and their own emotional well-being can contribute to their satisfaction and support. Brighton et al. [87] recommended acknowledging carers in breathlessness services, actively involving them and recognising them and their needs.

However, there are only a few interventions that specifically focus on addressing carers’ burden. This systematic review showed that carers mainly have a supportive role, such as always being present, managing extreme situations of breathlessness, being available all times during the day and night, listening carefully to patients´ needs, being selfless by giving up their own needs for social interaction, administering medications, and always bringing joy and happiness into patients’ life [88].

This systematic review confirms previous observations on gender inequality. Care work remains associated with females [89]. On average, in the European Union, 59% of all carers aged 18 years or over are identified as females [90]. No studies could be found that considered non-binary carers.

## Recommendations for practice

Patients with chronic diseases and breathlessness are particularly dependent on a good support network, including neighbourliness and mutual reciprocity, because breathlessness restricts their radius of movement. Particularly, in the case of breathlessness as a symptom complex that includes both physical symptoms and the psychological component of anxiety, an individual care network around the patient´s home is essential for good care. Expanding the scope of such care networks could also reduce the burden on carers, therefore we hypothesise that the establishment of neighbourliness at the societal level might be one strategy to support the carers. However, this must to be proven and evaluated in future studies. In addition, this strategy is probably not breathlessness specific and applies not only to carers of breathless patients. Based on the evidence of included studies, it seems worth evaluating.

Existing trends (development of intersectoral care, aging population, and increasing shortages of skilled workers) in healthcare require new approaches to healthcare delivery and its sustainability [90]. Carers are highly relevant resource that should be protected and supported. The concept of empowerment in community-building should be further expanded, and socio-political support structures should also be planned, such as providing instrumental support in the form of finances and improving health promotion among carers.

From a clinical perspective, the carer is primarily seen as a supporter of the patient’s well-being. Less attention is paid to the carers themselves and their feelings of anxiety and helplessness. They are expected to successfully shoulder responsibility. In critical situations, they are responsible for deciding when to seek help or administer oxygen and for determining the dosage. Healthcare professionals should be more aware of the carers’ burden caused by the high level of responsibility in caring for patients with breathlessness.

From the individual perspective, carers become increasingly concerned about their care recipients, while patients worry more about their carers than about their own well-being. It would be beneficial to develop holistic support approaches for carers to alleviate the concerns of both carers and patients.

Preparing carers for emergencies and providing clear instructions and information are resource-saving interventions on all three sides (carers, patients, and the healthcare system). This could give carers confidence in providing care and in breathlessness situations. If they feel safe, they could be better cared for at home with a higher chance of more time of good quality. They may spend more qualitative time with their patients, which enables a better balance between care tasks and personal well-being.

## Recommendations for research

This systematic search revealed that the lack of consistent wording and concepts regarding breathlessness poses a hurdle for scientific advancement. Therefore, establishing uniform concepts in wording and keywords is urgently recommended.

Furthermore, there was rarely a clear separation of data relating to patients and carers, rendering it difficult to extract information relating only to carers’ burden, (unmet) needs, and coping strategies due to breathlessness. The existence of the carer-patient dyad and its interactive situation can be considered; alternatively, a separate focus on carers would be beneficial for the development of targeted support services. In addition, improvement in the documentation and reporting of carers’ involvement in interventional studies would enhance researchers’ and clinicians´ understanding of and ability to assess the effectiveness of interventions in supporting and addressing carers´ needs.

In addition, health economic evaluation studies would be helpful in analysing the financing of long-term care services by carers within the insurance sector, because care for chronically ill individuals is a societal responsibility that is not achievable through professional nursing care alone.

## Strengths and limitations

These findings should be considered in relation to several limitations. First, the search process was considerably more challenging than, for example, a review of literature on a particular medication due to the lack of standardised terminology and definitions of terms and keywords. This resulted in an extremely diverse range of studies that needed to be carefully synthesised. Because a lot of information about carers was hidden in tables and appendices, a deep search was necessary for our results. Despite the heterogeneity of the studies, we successfully extracted, systematised, and analysed all the results, providing a comprehensive overview of both qualitative and quantitative data for carers of patients with breathlessness.

Second, the decision to include all studies, regardless of their quality and methodological focus, might have impacted the findings and strength of the evidence. However, this approach was necessary to obtain a comprehensive overview of the current state of the research. Carers are often discussed in relation to patient studies, and by including a wide range of studies, it was possible to gain a broader understanding of the carers’ phenomenon.

Finally, the analysis of interventional studies in relation to carers was challenging because of incomplete descriptions of their involvement. This lack of clarity and detail regarding carers’ roles limits our ability to effectively classify and categorise interventions. Nonetheless, this novel study compiled diverse studies, extracting and analysing different forms and methodologies, to achieve a broader comprehension of the challenges, needs, and management of carers for patients with breathlessness. Through high-level collaboration and teamwork, we have effectively delivered an in-depth and inclusive systematic review of all the available evidence.

## Conclusion

Overall, our findings highlight the significant emotional burden experienced by carers, especially the heightened anxiety and constant vigilance associated with breathlessness. It also provides valuable insights by establishing a connection between unmet needs and increased breathlessness in carers. Furthermore, our findings suggest that carer-specific breathlessness interventions may contribute to improved symptom management among patients, and thereby improving the overall well-being of both patients and carers. Improving carers´ confidence in care work can reduce carers´ anxiety.

Existing interventions tended to prioritise the patient's symptoms (breathlessness) without adequately addressing the overall burden and needs of carers. Future intervention development should aim to reflect on the experiences and needs of carers of patients with breathlessness. A comprehensive and holistic intervention in the area of caregiving for carers of patients with breathlessness would be valuable. Such interventions should aim to provide immediate support for the challenges associated with breathlessness and empower carers to prepare for life after caregiving. Because carers are extremely burden due to patient’s breathlessness. They describe unmet needs and uses individual coping strategies.

## Supplementary Information

Supplementary Material 1.

## Data Availability

The data used to support the findings of this study are available from the corresponding author upon request.
