# Supplementary material for: BreathCarer: Informal carers of patients with chronic breathlessness: a mixed-methods systematic review of burden, needs, coping, and support interventions
Source: BMC Palliat Care. 2025 Feb 1;24:33. doi: 10.1186/s12904-025-01670-0 (PMC11786361; doi:10.1186/s12904-025-01670-0)
Supplement: Supplementary file 1 — Supplementary Material 1. [file 12904_2025_1670_MOESM1_ESM.docx]

**Supplement I: Search Strategies**

| **MEDLINE (OVID)** | |
| --- | --- |
| #1 | exp Caregivers/ |
| #2 | exp Family/ |
| #3 | exp Home Nursing/ |
| #4 | (caregiver* or care giver* or carer* or home nursing).mp. [mp=title, abstract, original title, name of substance word, subject heading word, floating sub-heading word, keyword heading word, organism supplementary concept word, protocol supplementary concept word, rare disease supplementary concept word, unique identifier, synonyms] |
| #5 | ((family or families or spouse* or husband* or wife or wives or partner* or parent* or grandparent* or sibling* or mother* or father* or daughter* or son* or brother* or sister* or relative* or friend* or close person* or significant other* or child or children or loved one* or non-professional*) adj3 (care* or caring)).mp. [mp=title, abstract, original title, name of substance word, subject heading word, floating sub-heading word, keyword heading word, organism supplementary concept word, protocol supplementary concept word, rare disease supplementary concept word, unique identifier, synonyms] |
| #6 | 1 or 2 or 3 or 4 or 5 |
| #7 | exp dyspnea/ |
| #8 | (dyspn* or breathless*).mp. [mp=title, abstract, original title, name of substance word, subject heading word, floating sub-heading word, keyword heading word, organism supplementary concept word, protocol supplementary concept word, rare disease supplementary concept word, unique identifier, synonyms] |
| #9 | ((short* or labor* or labour* or difficult*) adj3 (breath* or respirat*)).mp. [mp=title, abstract, original title, name of substance word, subject heading word, floating sub-heading word, keyword heading word, organism supplementary concept word, protocol supplementary concept word, rare disease supplementary concept word, unique identifier, synonyms] |
| #10 | 7 or 8 or 9 |
| #11 | 6 and 10 |
| **PsycINFO (EbscoHost)** | |
| S1 | DE "Caregivers" |
| S2 | DE "Family" |
| S3 | TX (caregiver* or care giver* or carer* or home nursing) |
| S4 | TX ((family or families or spouse* or husband* or wife or wives or partner* or parent* or grandparent* or sibling* or mother* or father* or daughter* or son* or brother* or sister* or relative* or friend* or close person* or significant other* or child or children or loved one* or non-professional*) and (care* or caring)) |
| S5 | S1 OR S2 OR S3 OR S4 |
| S6 | DE "Dyspnea" |
| S7 | TX (dyspn* or breathless*) |
| S8 | TX ((short* or labor* or labour* or difficult*) N3 (breath* or respirat*)) |
| S9 | S6 OR S7 OR S8 |
| S10 | S5 AND S9 |
| **CINAHL (EbscoHost)** | |
| S1 | (MH "Caregivers") |
| S2 | (MH "Caregiver Burden") |
| S3 | (MH "Caregiver Support") |
| S4 | (MH "Family") |
| S5 | (MH "Home Nursing") |
| S6 | TI (caregiver* or care giver* or carer* or home nursing) |
| S7 | AB (caregiver* or care giver* or carer* or home nursing) |
| S8 | TI ((family or families or spouse* or husband* or wife or wives or partner* or parent* or grandparent* or sibling* or mother* or father* or daughter* or son* or brother* or sister* or relative* or friend* or close person* or significant other* or child or children or loved one* or non-professional*) N3 (care* or caring)) |
| S9 | AB ((family or families or spouse* or husband* or wife or wives or partner* or parent* or grandparent* or sibling* or mother* or father* or daughter* or son* or brother* or sister* or relative* or friend* or close person* or significant other* or child or children or loved one* or non-professional*) N3 (care* or caring)) |
| S10 | S1 OR S2 OR S3 OR S4 OR S5 OR S6 OR S7 OR S8 OR S9 |
| S11 | (MH "Dyspnea") |
| S12 | TI (dyspn* or breathless*) |
| S13 | AB (dyspn* or breathless*) |
| S14 | TI ((short* or labor* or labour* or difficult*) N3 (breath* or respirat*)) |
| S15 | AB ((short* or labor* or labour* or difficult*) N3 (breath* or respirat*)) |
| S16 | S11 OR S13 OR S14 OR S15 |
| S17 | S10 AND S16 |
| **CENTRAL** | |
| #1 | MeSH descriptor: [Caregivers] explode all trees |
| #2 | MeSH descriptor: [Family] explode all trees |
| #3 | (caregiver* or care giver* or carer* or home nursing) ti,ab,kw |
| #4 | ((family or families or spouse* or husband* or wife or wives or partner* or parent* or grandparent* or sibling* or mother* or father* or daughter* or son* or brother* or sister* or relative* or friend* or close person* or significant other* or child or children or loved one* or non-professional*) and (care* or caring)) ti,ab,kw |
| #5 | #1 OR #2 OR #3 OR #4 |
| #6 | MeSH descriptor: [Dyspnea] explode all trees |
| #7 | (dyspn* or breathless*) ti,ab,kw |
| #8 | ((short* or labor* or labour* or difficult*) adj3 (breath* or respirat*)) |
| #9 | #6 OR #7 OR #7 OR #8 |
| #10 | #5 AND #9 in Trials |

# Supplement II: Excluded Studies with Reasons

| **Author, Year** | **Reason** |
| --- | --- |
| Aasbo, Solbraekke et al. 2016 | No informal carer outcome specifically related to breathlessness |
| Addington-Hall and McCarthy 1995 | No informal carer outcome specifically related to breathlessness |
| Addington-Hall and O'Callaghan 2009 | No informal carer outcome specifically related to breathlessness |
| Adelman, Albert et al. 2004 | No informal carer outcome specifically related to breathlessness |
| Ågren, Berg et al. 2014 | Wrong population |
| Ågren, Evangelista et al. 2012 | Wrong population |
| Allen, Hilgeman et al. 2008, | Wrong Population |
| Andersen, Thomsen et al. 2017 | No informal carer outcome specifically related to breathlessness |
| Andersen, Thomsen et al. 2018 | No informal carer outcome specifically related to breathlessness |
| Andreou, Dhand et al. 2022 | Wrong population |
| Ängerud, Boman et al. 2018 | Wrong Population |
| Apps, Harrison et al. 2017 | No informal carer outcome specifically related to breathlessness |
| Archibald, Bakal et al. 2021 | No informal carer outcome specifically related to breathlessness |
| Ates, Ebenau et al. 2018 | No informal carer outcome specifically related to breathlessness |
| Bailey 2001 | No informal carer outcome specifically related to breathlessness |
| Baillie, Anagnostou et al. 2018, | No informal carer outcome specifically related to breathlessness |
| Bajwah, Higginson et al. 2013, | Results not clearly related to informal carer |
| Bakas, Lewis et al. 2001 | No informal carer outcome specifically related to breathlessness |
| Barnes, Gott et al. 2006 | No informal carer outcome specifically related to breathlessness |
| Barnes-Harris, Daniel et al. 2021 | Wrong Population |
| Bartlett, Webb et al. 2017 | No informal carer outcome specifically related to breathlessness |
| Bebb, Murray, et al. 2023 | Wrong population |
| Bell-Davies, Goyder et al. 2019 | No informal carer outcome specifically related to breathlessness |
| Benson, Washington et al. 2021 | No informal carer outcome specifically related to breathlessness |
| Bidwell, Higgins et al. 2018 | No informal carer outcome specifically related to breathlessness |
| Bischoff, Choi et al. 2021 | No informal carer outcome specifically related to breathlessness |
| Blanck, Fors et al. 2021 | No informal carer outcome specifically related to breathlessness |
| Bove, Zakrisson et al. 2016 | No informal carer outcome specifically related to breathlessness |
| Boyle 2009 | No informal carer outcome specifically related to breathlessness |
| Braun, Hales et al. 2012 | Wrong population |
| Braun, Mikulincer et al. 2007 | Wrong population |
| Brazil, Kaasalainen et al. 2013 | Wrong population |
| Brown and Cohen 2020 | Wrong population |
| Bruley 2003 | Full text not available |
| Buck, Benitez et al. 2020 | No informal carer outcome specifically related to breathlessness |
| Buckingham, Kendall et al. 2015 | No informal carer outcome specifically related to breathlessness |
| Burge, Lawson et al. 2014 | Wrong Population |
| Burke, Hardiman et al. 2018 | No informal carer outcome specifically related to breathlessness |
| Burke, Johnson-Koenke et al. 2016 | No informal carer outcome specifically related to breathlessness |
| Caga, Hsieh et al. 2018 | Wrong population |
| Caggianelli, Iovino et al. 2022 | No informal carer outcome specifically related to breathlessness |
| Campbell 2014 (Letter to the Editor) | No informal carer outcome specifically related to breathlessness |
| Caprio, Hanson et al. 2008 | No informal carer outcome specifically related to breathlessness |
| Caress, Luker et al. 2010 | No informal carer outcome specifically related to breathlessness |
| Chellappan, Ezhilarasu et al. 2014 | No informal carer outcome specifically related to breathlessness |
| Chiò, Gauthier et al. 2005 | Wrong population |
| Cloyes, Thomas Hebdon et al. 2023 | No informal carer outcome specifically related to breathlessness |
| Comini, Rocchi et al. 2016 | Wrong Population |
| Cooney, Proulx et al. 2021 | No informal carer outcome specifically related to breathlessness |
| Costa, Gomez-Batiste et al. 2016 | No informal carer outcome specifically related to breathlessness |
| Costantini, Pellegrini et al. 2014 | No informal carer outcome specifically related to breathlessness |
| Cox, Wilson et al. 2006 | Wrong Population |
| Currow, Ward et al. 2008 | No informal carer outcome specifically related to breathlessness |
| Dalal, Taylor et al. 2021 | No informal carer outcome specifically related to breathlessness |
| den Herder-van der Eerden, Hasselaar et al. 2017 | Wrong population |
| Dionne-Odom, Ejem et al. 2020 | Wrong population |
| Dionne-Odom, Wells et al. 2022 | Wrong population |
| Dismore, Echevarria et al. 2019 | No informal carer outcome specifically related to breathlessness |
| Drury, Goss et al. 2023 | No informal carer outcome specifically related to breathlessness |
| Durante, Paturzo et al. 2019 | No informal carer outcome specifically related to breathlessness |
| Ek, Andershed et al. 2015 | No informal carer outcome specifically related to breathlessness |
| Ellis, Wagland et al. 2012 | No informal carer outcome specifically related to breathlessness |
| Ellis, Warden et al. 2017 | No informal carer outcome specifically related to breathlessness |
| Ewing, Penfold et al. 2017 | Wrong Population |
| Farquhar, Higginson et al. 2009 | No informal carer outcome specifically related to breathlessness |
| Ferreira, Boland et al. 2020 | No informal carer outcome specifically related to breathlessness |
| Ferrell, Ruel et al. 2022 | No informal carer outcome specifically related to breathlessness |
| Figueiredo, Gabriel et al. 2014 | Wrong population |
| Fitch 2020 | No informal carer outcome specifically related to breathlessness |
| Galatsch, Prigerson et al. 2019 | Wrong population |
| Gallagher, Lagman et al. 2006 | Wrong Population |
| Gallagher, Lagman et al. 2017 | No informal carer outcome specifically related to breathlessness |
| Garcia, Luckett et al. 2019 | No informal carer outcome specifically related to breathlessness |
| Gautun, Werner et al. 2012 | No informal carer outcome specifically related to breathlessness |
| Gazzi, Comini et al. 2022 | Wrong population |
| Given, Stommel et al. 1993 | Wrong population |
| Goodfellow, Stark et al. 2020 | Wrong Population |
| Götze, Brähler et al. 2014 | No informal carer outcome specifically related to breathlessness |
| Grant, Sun et al. 2013 | No informal carer outcome specifically related to breathlessness |
| Greaves, Wingham et al. 2016, | No informal carer outcome specifically related to breathlessness |
| Gysels and Higginson 2009 | Results not clearly related to informal carer |
| Harding, Higginson et al. 2003 | No informal carer outcome specifically related to breathlessness |
| Haun, Sklenarova et al. 2014 | Wrong population |
| Higginson and Gao 2008 | No informal carer outcome specifically related to breathlessness |
| Higginson, Bausewein et al. 2014 | No informal carer outcome specifically related to breathlessness |
| Hipólito, Ruivo et al. 2020 | No informal carer outcome specifically related to breathlessness |
| Hoenig, van der Laan et al. 2023 | No informal carer outcome specifically related to breathlessness |
| Holden, Schubert et al. 2015 | Wrong Population |
| Holm, Bowler et al. 2009 | No informal carer outcome specifically related to breathlessness |
| Horton 2012 | No informal carer outcome specifically related to breathlessness |
| Hsu, Wu et al. 2021 | No informal carer outcome specifically related to breathlessness |
| Hutchinson, Galvin et al. 2020 | Results not clearly related to informal carer |
| Hutchinson, Pickering et al. 2017 | No informal carer outcome specifically related to breathlessness |
| Ivziku, Clari et al. 2019 | Wrong Population |
| Jeong and Yoo 2015 | No informal carer outcome specifically related to breathlessness |
| Johnson, Booth et al. 2016 | unsuccessful author contact |
| Johnson, Kassner et al. 2005 | No informal carer outcome specifically related to breathlessness |
| Johnson, McSkimming et al. 2018 | No informal carer outcome specifically related to breathlessness |
| Jonsdottir 2007 | No informal carer outcome specifically related to breathlessness |
| Jonsdottir and Ingadottir 2011, | No informal carer outcome specifically related to breathlessness |
| Jonsdottir, Amundadottir et al. 2015 | No informal carer outcome specifically related to breathlessness |
| Kaasalainen, Strachan et al. 2011 | No informal carer outcome specifically related to breathlessness |
| Kalluri, Younus et al. 2021 | No informal carer outcome specifically related to breathlessness |
| Kanervisto, Kaistila et al. 2007 | No informal carer outcome specifically related to breathlessness |
| Kang, Keam et al. 2021 | No informal carer outcome specifically related to breathlessness |
| Kazanowski 2005 | Wrong Population |
| Kehl 2007 | Full text not available |
| Kin, Tsang et al. 2021 | No informal carer outcome specifically related to breathlessness |
| Knowles, Combs et al. 2016 | Wrong population |
| Kozachik, Given et al. 2001 | Wrong population |
| Krishnasamy, Wilkie et al. 2001 | No informal carer outcome specifically related to breathlessness |
| Kristanti, Setiyarini et al. 2017 | No informal carer outcome specifically related to breathlessness |
| Lam, Harding et al. 2023 | No informal carer outcome specifically related to breathlessness |
| Lang, Smith et al. 2018 | No informal carer outcome specifically related to breathlessness |
| LeBon and Fisher 2011 | Wrong Population |
| Lee, Lum et al. 2010 | Wrong Population |
| Lee, Mudd et al. 2017 | No informal carer outcome specifically related to breathlessness |
| Lian, Zheng et al. 2022 | Wrong population |
| Lindqvist, Albin et al. 2013 | No informal carer outcome specifically related to breathlessness |
| Lopez, Chaoul et al. 2018 | No informal carer outcome specifically related to breathlessness |
| Lynn, Ely et al. 2000 | No informal carer outcome specifically related to breathlessness |
| Lyons and Lee 2020 | Wrong population |
| Lyons, Miller et al. 2016 | Wrong population |
| MacKenzie, Buck et al. 2016 | No informal carer outcome specifically related to breathlessness |
| Magnani, Lenoci et al. 2017 | No informal carer outcome specifically related to breathlessness |
| Marques, Gabriel et al. 2015 | Wrong Population |
| Marques, Jácome et al. 2015 | No informal carer outcome specifically related to breathlessness |
| Marques, Jácome et al. 2019 | Wrong Population |
| McDermott, Bradburn et al. 2016 | No informal carer outcome specifically related to breathlessness |
| McDonnell, Gallerani et al. 2020 | Wrong Population |
| McEvoy, Scott et al. 2018 | No informal carer outcome specifically related to breathlessness |
| McHorney, Mansukhani et al. 2021 | No informal carer outcome specifically related to breathlessness |
| McMillan and Moody 2003 | No informal carer outcome specifically related to breathlessness |
| McMillan and Small 2007 | No informal carer outcome specifically related to breathlessness |
| Meier, Bodenmann et al. 2011 | No informal carer outcome specifically related to breathlessness |
| Meier, Mörgeli et al. 2011 | No informal carer outcome specifically related to breathlessness |
| Milbury, Liao et al. 2019 | Wrong Population |
| Miravitlles, Pena-Longobardo et al. 2015 | Wrong population |
| Mishra, Gupta et al. 2022 | No informal carer outcome specifically related to breathlessness |
| Mishra, Gupta et al. 2022 | No informal carer outcome specifically related to breathlessness |
| Mosher, Ott et al. 2015 | No informal carer outcome specifically related to breathlessness |
| Mosher, Secinti et al. 2019 | Wrong Population |
| Murphy, Felgoise et al. 2009 | Wrong population |
| Mustfa, Walsh et al. 2006 | Wrong Population |
| Nguyen, Khanh et al. 2023 | No informal carer outcome specifically related to breathlessness |
| Niu, Guo et al. 2021 | Wrong population |
| Olesen, la Cour et al. 2022 | No informal carer outcome specifically related to breathlessness |
| Overgaard, Kaldan et al. 2016 | No informal carer outcome specifically related to breathlessness |
| Ozcelik, Fadiloglu et al. 2014 | Wrong Population |
| Petruzzo, Biagioli et al. 2019 | No informal carer outcome specifically related to breathlessness |
| Phongtankuel, Teresi et al. 2020 | No informal carer outcome specifically related to breathlessness |
| Piamjariyakul, Smith et al. 2013 | No informal carer outcome specifically related to breathlessness |
| Piamjariyakul, Werkowitch et al. 2015 | No informal carer outcome specifically related to breathlessness |
| Piette, Striplin et al. 2015 | No informal carer outcome specifically related to breathlessness |
| Porter, Keefe et al. 2011 | unsuccessful author contact |
| Pressler, Gradus-Pizlo et al. 2013 | No informal carer outcome specifically related to breathlessness |
| Rehman, Muhammed et al. 2021 | No informal carer outcome specifically related to breathlessness |
| Sampson, Gill et al. 2015 | Wrong population |
| Schunk, Berger et al. 2021 | No informal carer outcome specifically related to breathlessness |
| Seamark, Blake et al. 2004 | No informal carer outcome specifically related to breathlessness |
| Sellner-Pogany and Lahrmann 2009 | No informal carer outcome specifically related to breathlessness |
| Sharafkhaneh, Wolf et al. 2013 | No informal carer outcome specifically related to breathlessness |
| Simpson, Young et al. 2010 | No informal carer outcome specifically related to breathlessness |
| Smothers and Buck 2012 | Full text not available |
| Souto-Miranda and Marques 2019 | No informal carer outcome specifically related to breathlessness |
| Spence, Hasson et al. 2008 | No informal carer outcome specifically related to breathlessness |
| Stevens and Abrahm 2019 | No informal carer outcome specifically related to breathlessness |
| Strang, Fährn et al. 2019 | No informal carer outcome specifically related to breathlessness |
| Sun, Grant et al. 2015, | No informal carer outcome specifically related to breathlessness |
| Sun, Raz et al. 2017 | No informal carer outcome specifically related to breathlessness |
| Suresh, Young et al. 2022 | No informal carer outcome specifically related to breathlessness |
| Teno, Clarridge et al. 2004 | No informal carer outcome specifically related to breathlessness |
| Thomas, Warrier et al. 2018 | Wrong population |
| Trail, Nelson et al. 2003 | Wrong population |
| Utens, van Schayck et al. 2014, | No informal carer outcome specifically related to breathlessness |
| Wagland, Ellis et al. 2012 | Wrong population |
| Wakabayashi, Motegi et al. 2011 | No informal carer outcome specifically related to breathlessness |
| Weitzner, Moody et al. 1997 | No informal carer outcome specifically related to breathlessness |
| White, White et al. 2011 | No informal carer outcome specifically related to breathlessness |
| Wieland, Hoppe et al. 2019 | No informal carer outcome specifically related to breathlessness |
| Xu, Zheng et al. 2022 | No informal carer outcome specifically related to breathlessness |
| Zeb, Younas et al. 2021 | Wrong population |
| Zhang, Sharma et al. 2022 | No informal carer outcome specifically related to breathlessness |
| Bausewein, Jolley et al. 2012  Byrne, Sampson et al. 2013  Farquahr, Prevost et al. 2011  Nakkan, Janssen et al. 2014  Piamjariyakul, Smothers et al. 2020  [https://clinicaltrials.gov/show/NCT00711438 2008](https://clinicaltrials.gov/show/NCT00711438%202008)  [https://clinicaltrials.gov/show/NCT00678405 2008](https://clinicaltrials.gov/show/NCT00678405%202008)  [https://clinicaltrials.gov/show/NCT02048306 2014](https://clinicaltrials.gov/show/NCT02048306%202014)  [https://clinicaltrials.gov/show/NCT02622412 2015](https://clinicaltrials.gov/show/NCT02622412%202015)  <https://clinicaltrials.gov/show/NCT03813667> 2019 | Protocols of completed studies and Trial Registrations |

# Supplement III: Characteristics of ongoing studies

| **Study** | **Population** | **Intervention** | **Outcome** |
| --- | --- | --- | --- |
| **Ongoing trials** | | | |
| Actrn (2021). "A phase 3 clinical trial examining the effects of SingINg For breathing in chronic obstructive pulmonary disease (COPD) aNd interstitial lung disease (ILD) pAtients."  <https://trialsearch.who.int/Trial2.aspx?TrialID=ACTRN12621001280897> | Patient Cohort:   - Diagnosis of COPD or ILD - Modified Medical Research Council dyspnoea score grerater than or equal to 2 - Aged 18 years or over   Carers Cohort (optional):   - Nominated carers of eligible patients will be eligible to participate. - Aged 18 years or over | Phase 3 is a double-arm, randomised, analysis, mixed-methods intervention study of online singing group classes compared to standard care for participants with lung disease.  Arm 1: Intervention – Participation in an online group singing class each week for 12 weeks.  Arm 2: Control – 12 weeks of standard of care. Participants are telephoned monthly to complete study questionnaires. Control arm participants will have the option of enrolling in 12-weeks of free online singing group classes at the completion of the study. No data will be collected for that period.  Carer enrolment is optional for both arms of both phases. If enrolled, carers will have the option to participate in singing groups and additional singing at home exercises. | Phase 3 (efficacy outcome)   1. Quality of life measured using the SF-36 2. Carer quality of life measured on the CareQOL and SF-36 3. Breathlessness measured using the Dyspnoea-12 4. Breathlessness measured using the mastery subdomain of the CRQ 5. Depression and anxiety measured using the HADS scale 6. Healthcare utilisation measured by self-reported healthcare utilisation questionnaire designed for this study 7. Loneliness measured using the UCLA-3 short form |
| Actrn (2021). "A phase 2 clinical trial examining the feasibility of SingINg For breathing in chronic obstructive pulmonary disease (COPD) aNd interstitial lung disease (ILD) pAtients."  <https://trialsearch.who.int/Trial2.aspx?TrialID=ACTRN12621001274864> | Patient Cohort:   - Diagnosis of COPD or ILD - Modified Medical Research Council dyspnoea score grerater than or equal to 2 - Aged 18 years or over   Carers Cohort (optional):   - Nominated carers of eligible patients will be eligible to participate.   Aged 18 years or over | Phase 2 is a double-arm, randomised, blinded-analysis, mixed-methods feasibility study of online singing group classes compared to standard care for participants with lung disease.  Arm 1: Intervention – Participation in an online group singing class each week for 12 weeks.  Arm 2: Control – 12 weeks of standard of care. Participants are telephoned monthly to complete study questionnaires. Control arm participants will have the option of enrolling in 12-weeks of free online singing group classes at the completion of the study. No data will be collected for that period.  Carer enrolment is optional for both arms of both phases. If enrolled, carers will have the option to participate in singing groups and additional singing at home exercises. | Phase 2 (feasibility outcome)   1. Breathlessness measured using the Dyspnoea-12 2. Breathlessness measured using the mastery subdomain of the CRQ 3. Carer quality of life measured on the CareQOL and SF-36 4. Depression and anxiety measured using the HADS scale 5. Healthcare utilisation measured by self-reported healthcare utilisation questionnaire designed for this study at monthly intervals 6. Loneliness measured using the UCLA-3 short form |
| Isrctn (2017). "Working together against COPD."  <https://trialsearch.who.int/Trial2.aspx?TrialID=ISRCTN59537391> | Patients: Adults with a confirmed diagnosis of COPD  Carers: Identified by a participant with COPD in the study as a ‘particular family carer or friend who helps them. | Following patient recruitment and collection of baseline data, participants will be randomised to the TANDEM intervention or usual care (control) using minimisation with a random element: this will be done in order to minimise potential imbalances at baseline for anxiety (HADS-A), depression (HADS-D), dyspnoea (mMRC) and smoking.  TANDEM (Intervention group): Participants will receive weekly, one-to-one cognitive behavioural approach (CBA) sessions by a TANDEM trained respiratory health care professionals prior to commencement of pulmonary rehabilitation. Weekly phone calls will continue whilst the participant is attending pulmonary rehabilitation and for 2 weeks after its completion.  Usual care (Control group): Participants in the control arm will follow local arrangements for the provision of pulmonary rehabilitation. Participants will be offered the British Lung Foundation DVD on living with COPD and booklets on COPD and pulmonary rehabilitation. | Primary outcome: Anxiety and depression are measured using the Hospital Anxiety and Depression Scale (HADS)  Secondary Outcome:  Patient outcomes:   1. Degree of breathlessness is measured by the modified Medical Research Council (mMRC) Breathlessness scale 2. Depression is measured using the Beck’s Depression Inventory-II (BDI-II) 3. Anxiety is measured using the Beck’s Anxiety Inventory (BAI) 4. Respiratory health-related quality of life is measured using the St George's Respiratory Questionnaire (SGRQ) 5. Illness perception about COPD is measured using the Brief-Illness Perception Questionnaire (B-IPQ) 6. Social engagement is measured using the Health Education Impact Questionnaire (heiQ) 7. Functional/social activity is measure using the Time Use Survey   Carer outcomes:   1. Carer burden is measured using the Zarit Burden Interview (ZBI) 2. Carer mental well-being is measured using the Warwick-Edinburgh Mental Wellbeing Scale (WEMWBS) |
| Isrctn (2019). "BETTER-B: Better treatments for persistent breathlessness."  <https://trialsearch.who.int/Trial2.aspx?TrialID=ISRCTN10487976> | Patients: Aged 18 years old  Diagnosed with COPD, ILD, including chronic fibrotic lung disease following SARS-CoV-2 infection Breathlessness severity: Modified MRC breathlessness scale  Carers: Identified by an included participant as the person closest to them, aged ≥18 years old, able to complete questionnaires and assessments and to provide written informed consent | Participants with refractory breathlessness and COPD/ILD will be randomised on a 1:1 basis to receive either oral Mirtazapine or Placebo. | Primary Outcome: Self-reported worst breathlessness over the past 24 hours measured at day 56 post start of treatment using a numerical rating scale (NRS, 0=no breathlessness to 10=worst possible breathlessness)  Secondary Outcome  Different outcomes for patients: e.g.  Number and duration of episodes of breathlessness over the last 24 hours,  Physical and emotional aspects of breathlessness (Dyspnoea, fatigue, emotional function, mastery) as assessed by the Chronic Respiratory Questionnaire (CRQ)  Physical symptoms as assessed by the Integrated Palliative care Outcome Scale (IPOS), Quality of Life (QoL) as assessed by the EQ-5D-5L and associated VAS and Australia-modified Karnofsky Performance Scale (AKPS)  Formal and informal care use over the previous period as measured by the Client Services Receipt Inventory (CSRI  Carer Outcomes:  Carers assessment of the participant’s number and duration of episodes of breathlessness over the last 24 hours Carers self-reported burden as measured by the Zarit Burden inventory  Carers self-reported experiences of caregiving as measured by the Positive Aspects of Caregiving Scale (PAC)  Carers perspectives on participants’ situation as measured by the Integrated Palliative care outcome scale (IPOS)  Carers overall health and wellbeing as measured by EQ-5D-5L and associated VAS |
| Isrctn (2020). "BETTER-B (Aus): Better treatments for refractory breathlessness."  <https://trialsearch.who.int/Trial2.aspx?TrialID=ISRCTN15751764> | Patients: Aged 18 years old  Diagnosed with COPD, ILD, including chronic fibrotic lung disease following SARS-CoV-2 infection Breathlessness severity: Modified MRC breathlessness scale  Carers: Identified by an included participant as the person closest to them, aged ≥18 years old, able to complete questionnaires and assessments and to provide written informed consent | Participants will be randomised via minimisation in a 1:1 ratio to receive either oral mirtazapine or placebo medication for 56 days.  Carer part of study:  There will be no separate randomisation process for carers as they will be identified by the same trial number as the randomised participant. | Primary Outcome: Self-reported worst breathlessness over the last 24 hours at day 56 post start of treatment as assessed by numerical rating scale (NRS, 0=no breathlessness to 10=worst possible breathlessness)  Secondary Outcome  Different outcomes for patients: e.g.  Number and duration of episodes of breathlessness over the last 24 hours,  Physical and emotional aspects of breathlessness (Dyspnoea, fatigue, emotional function, mastery) as assessed by the Chronic Respiratory Questionnaire (CRQ)  Physical symptoms as assessed by the Integrated Palliative care Outcome Scale (IPOS), Quality of Life (QoL) as assessed by the EQ-5D-5L and associated VAS and Australia-modified Karnofsky Performance Scale (AKPS)  Formal and informal care use over the previous period as measured by the Client Services Receipt Inventory (CSRI)  Carer Outcomes:  Carers assessment of the participant’s number and duration of episodes of breathlessness over the last 24 hours Carers self-reported burden as measured by the Zarit Burden inventory  Carers self-reported experiences of caregiving as measured by the Positive Aspects of Caregiving Scale (PAC)  Carers perspective on participants’ situation as measured by the Integrated Palliative care outcome scale (IPOS)  Carer overall health and wellbeing as measured by EQ-5D-5L and associated VAS |
| Isrctn (2019). "Morphine and BrEathLessness trial (MABEL)."  <https://trialsearch.who.int/Trial2.aspx?TrialID=ISRCTN87329095> | Patients:  Ambulant people with chronic breathlessness due to Chronic heart failure, COPD, ILD, post-COVID chronic breathlessness, Cancer. Breathlessness severity defined as modified Medical Research Council (mMRC) breathlessness scale grade 3 or 4. Male or female aged 18 years. | Randomised patients will receive either modified release morphine 5 mg capsules twice daily with docusate laxative 100 mg capsules twice daily (intervention arm 1) or placebo “5mg” morphine capsules twice daily and placebo docusate laxative capsules twice daily (intervention arm 2). | Primary Outcome: The amount of breathlessness at its most over the previous 24 h (worst breathlessness) measured at day 28 using a numerical rating scale (NRS) where 0 = no breathlessness and 10 = the most imaginable breathlessness).  Secondary Outcome:   1. Distress due to breathlessness using 0-10 NRS 2. Assessment of related symptoms using 0-10 NRS and quality of sleep (Epworth Sleepiness Scale); sleepiness using the Karolinska Sleepiness Scale (KSS) 3. Assessment of physical activity (daily steps Actigraphy monitor), performance status (Australia modified Karnofsky Performance Status (AKPS) and cognitive function (St Louis University Mental Status (SLUMS) 4. Quality of life (SF-12) 5. Health economic assessment (EQ5D; EQVAS; ICECAP; service utilisation) 6. Harms, including survival 7. Carer burden (Zarit 12; VOICES) 8. Opioid withdrawal: Subjective opioid withdrawal scale (SOWS) |
| NCT04898972  Mindfulness-based Intervention in COPD Dyads  <https://clinicaltrials.gov/ct2/show/NCT04898972> | Patients: 18 Years and older affected by COPD stage C and stage D. | Intervention: Mindfulness-based stress reduction intervention-> Patient-family carer dyads will take part in 8-week Mindfulness-based stress reduction intervention (MBSR).  Active Comparator: Patient-informal carer dyads will receive an informative booklet on stress reduction strategies. | Primary Outcomes:   1. Change from baseline in perceived stress measured by Perceived Stress Scale (PSS) 2. Change from baseline in anxiety level measured by Generalized Anxiety Disorder scale (GAD-7) 3. Change from baseline in depression measured by Patient Health Questionnaire (PHQ-9)   Secondary Outcomes:   1. Change from baseline in subjective quality of sleep measured by the Pittsburgh Sleep Quality Index (PSQI) 2. Change from baseline in mindfulness experience measured by Five Facet Mindfulness Questionnaire (FFMQ) at 8 and 16 weeks. 3. Change in health-related quality of life measured by Short Form 12 Health Survey (SF-12) 4. Change in impact of the COPD on patient's life measured by COPD Assessment Test (CAT) 5. Change in patient's dyspnea measured by the Modified Medical Research Council scale (mMRC) |
| NCT05106257  Optimizing Self-management COPD Treatment Through the American Lung Association Helpline  <https://www.clinicaltrials.gov/ct2/show/NCT05106257> | Patients: COPD, male or female, 40 years or older. | Behavioral: Self-management education (short)  Behavioral: Ground-based walking training  Behavioral: Inhaler training  Behavioral: (informal) Caregiver support  Behavioral: Self-management education (long) | Primary Outcomes:   1. Health-related quality of life   Secondary Outcomes:   1. COPD symptom burden measured by the COPD Assessment Test (CAT) 2. Self-management behaviours measured by the Patient Activation Measure (PAM-13) 3. Hospitalisation, measured with self-reported hospitalisation history. |
| NCT04739696  Developing a Virtual Stress Management Intervention for Spousal/Partnered Caregivers of Solid Tumor Cancer Patients.  <https://www.clinicaltrials.gov/ct2/show/NCT04739696> | Patients: primary diagnosis of solid tumor cancer at any stage that are within 12 weeks +/- of starting treatment, which includes either infusion chemo- or immunotherapy, oral targeted agents, or both.  Caregiver:  A primary caregiver for the patient with a diagnosis of solid tumor cancer. Spouse or partner of the patient for at least a year  Caregiver must live with patient  Must be available to fully participate in an intervention (Virtual-PEPRR or PepPal) if assigned  Must score 1 or greater on the PHQ-2 (depression) and/or GAD-2 (anxiety) during pre-screening.  Must be employed at the time of the patient's diagnosis for a minimum of 20 hours/week with plans to remain employed and working during their patient's treatment. | No Intervention: Caregiver Control  biomarker analysis; questionnaire administration; survey administration; treatment as usual  Experimental: Caregiver Intervention  biomarker analysis; questionnaire administration; survey administration; Psycho Education Paced Respiration and Relaxation (PEPRR), which includes virtual one-on-one psychoeducation and stress management intervention.  Experimental: Caregiver Self-Directed  biomarker analysis; questionnaire administration; survey administration; Pep-Pal web-accessible video modules of the psychoeducation and stress management intervention. | Primary Outcomes:   1. Caregiver: Change in Center for Epidemiological Studies Depression Scale (CESD)   Secondary Outcomes:   1. Caregiver: Change in Spielberger State-Trait Anxiety Inventory (STAI) [ 2. Caregiver: Change in Perceived Stress Scale (PSS) 3. Caregiver: Health Care Utilization measured by electronic health records and supplemented by Colorado All Payer Claims Data and self-report 4. Caregiver: Change in Adrenal Activity Over Time measured by Cortisol in hair will be used as a retrospective measure of activation of the hypothalamic pituitary adrenal axis. 5. Caregiver: Change in Caregiver Telomere Length, which was assessed as a measure of cellular aging in blood samples from participants. 6. Patient: Change in Center for Epidemiological Studies Depression Scale (CESD) 7. Patient: Change in Spielberger State-Trait Anxiety Inventory (STAI) 8. Patient: Change in Perceived Stress Scale (PSS) 9. Patient: Health Care Utilization [ Time Frame: Baseline, 12 months follow up] 10. Patient: Change in MD Anderson Symptom Inventory (MDASI) 11. Caregiver: Employment status 12. Caregiver: Job Satisfaction |
| NCT05040386  Nurse Coach-Led Early Palliative Care for Older Adults With COPD and Their Care Partners: The Project EPIC Pilot RCT  <https://clinicaltrials.gov/ct2/show/NCT05040386> | Patient: ≥60 years, COPD on routinely collected spirometry (FEV1/FVC <0.70 + FEV1<80%), Have a care partner who is willing to participate, defined as "a person who knows you [the patient] well and is involved in your medical care"; Severe breathlessness as defined by a documented modified Medical Research Council (mMRC)  Care partner: ≥18 years; Self-reporting as "an unpaid spouse or care partner, relative, or friend who knows [the patient] well and is involved in their medical care"; | Active Comparator: Usual COPD Care  Participants randomised to this arm will receive the standard of care for COPD.  Experimental: Intervention (ENABLE-COPD plus Usual COPD Care). Participants randomised to this arm will receive the experimental treatment for COPD (i.e. ENABLE-COPD plus usual COPD care). | Primary Outcomes:   1. Intervention Feasibility: We will define ≥80% completion of components as evidence of feasibility. 2. Trial and Survey Feasibility: Rates of screening, enrolment, retention, reasons for exclusion and refusals, and attrition. Survey completion rates will be measured, and survey feasibility will be defined as participants completing ≥80% of instruments. 3. Acceptability (Quantitative): Post-intervention survey on intervention acceptability with Likert scale 4. Acceptability (Qualitative): Post-intervention semi-structured, in-depth interviews by telephone   Secondary Outcomes:   1. UAB Life Space Assessment (LSA) 2. Chronic Respiratory Questionnaire (CRQ) 3. PROMIS Global Health 4. Montgomery Borgatta Caregiver Burden 5. Hospital Anxiety and Depression Scale (HADS) 6. Modified Telephone Interview for Cognitive Status (mTICS) 7. Katz Index of Activities of Daily Living 8. Lawton Instrumental Activities of Daily Living 9. Healthcare and palliative care utilization: Rates of emergency room visits, hospitalisations, intensive care unit admissions, imaging, procedures, palliative care, hospice, change in code status, advance directive completion, identified surrogate decision maker, goals of care documentation 10. De Jon Gierveld Loneliness Scale |
| **Completed trials but no results available yet** | | | |
| Isrctn (2017). "Working together against COPD." <https://trialsearch.who.int/Trial2.aspx?TrialID=ISRCTN59537391> | Patients: Adults with a confirmed diagnosis of COPD, post bronchodilator FEV1/FVC ratio <70%  Carers: Identified by a participant with COPD in the study as a ‘particular family caregiver or friend who helps them’ whom they would be happy for us to invite to join the study. | Intervention(s)  TANDEM intervention: Participants in the intervention arm will receive between 5 and 8 weekly, one-to-one cognitive behavioural approach (CBA) sessions by a TANDEM trained respiratory health care professionals (termed 'facilitators') prior to commencement of pulmonary rehabilitation.  Usual care (Control group): Receive local arrangements for the provision of pulmonary rehabilitation referred to the service (including any psychological treatment provided routinely in that service). In agreement with the local service, participants will be offered the British Lung Foundation DVD on living with COPD and additional booklets on COPD and pulmonary rehabilitation. | Measured all at baseline, 6-months, 12 months  Primary Outcome:  Anxiety and depression: Hospital Anxiety and Depression Scale (HADS) subscales (HADS-A and HADS-D)  Secondary Outcomes:  Patient outcomes:   1. Degree of breathlessness: modified Medical Research Council (mMRC) Breathlessness scale 2. Depression: Beck’s Depression Inventory-II (BDI-II) 3. Anxiety: Beck’s Anxiety Inventory (BAI) 4. Respiratory health-related quality of life: St George's Respiratory Questionnaire (SGRQ) 5. Illness perception about COPD: Brief-Illness Perception Questionnaire (B-IPQ) 6. Social engagement: Health Education Impact Questionnaire (heiQ) 7. Functional/social activity:Time Use Survey   Carer outcomes:   1. Carer burden: Zarit Burden Interview (ZBI) 2. Carer mental well-being: Warwick-Edinburgh Mental Wellbeing Scale (WEMWBS) |
| **Published Protocols (ongoing trials)** | | | |
| Protocol:  U. Piamjariyakul, T. Petitte, A. Smothers, S. Wen, E. Morrissey, S. Young, et al.  Study protocol of coaching end-of-life palliative care for advanced heart failure patients and their family caregivers in rural appalachia: a randomised controlled trial | Patients: Advanced HF (NYHA III or IV) | Intervention group: Intervention participants will receive standard care plus five weekly coaching sessions with telephone follow-up to reinforce HF palliative home care. Community-based nurses will deliver FamPALcare intervention. FamPALcare intervention involves coaching patients and family caregivers in advanced HF home care and supporting EOLPC discussions.  Control Group: The standard care group will receive routine HF care and instruction at university hospital or at clinic appointments. All patients can be referred for supportive care and heart failure care per national HF guidelines. | Primary Outcomes:   1. Patient HF health status measured by Kansas City Cardiomyopathy Questionnaire (KCCQ) over 6 months. KCCQ is a 12-item Likert scale (range 0-4). 2. Patient mental health - depression & anxiety measured by -item Likert Patient Health Questionnaire (PHQ-4) 3. Patient signed advance directive 4. Caregiver mental health - depression & anxiety measured by Patient Health Questionnaire (PHQ-4) 5. Caregiver quality of life - physical and mental health measured by SF12V2 Health Survey (PCS and MCS scores). 6. Caregiver burden measured by 12-item Likert Short-form Caregiver Burden Interviews 7. Patient and caregiver confidence in providing palliative home care for advanced HF measured by 4-item Likert Confidence scale 8. Patient and caregiver preparedness in providing palliative home care for advanced HF measured by 1-item Likert perceived preparedness question   Secondary Outcomes:   1. Evaluate intervention helpfulness (patients and caregivers) 2. Evaluate intervention helpfulness (healthcare professionals) 3. Evaluate healthcare utilizations |
| Caregiver-assisted coping skills training for patients with COPD: background, design, and methodological issues for the INSPIRE-II study  J. A. Blumenthal, F. J. Keefe, M. A. Babyak, V. C. Fenwick, J. M. Johnson, K. Stott, et al. (2009) | COPD; FEV1 30–80% of predicted value within 6 months of study enrolment, FEV1/FVC <70%, and having an identified caregiver. | RCT  Intervention: Enhanced Coping skills training (CST): to systematically train patients (and caregivers) in the use of coping skills for symptom management. Patients and caregivers will receive 14, 30-min telephone calls (12 weekly sessions followed by 2 bi-weekly booster sessions) over a 16-week period.  Control: Standardized care including COPD education and symptom monitoring. | Primary Outcomes:  (1) combined death and hospitalisations/COPD-related physician visits  (2) Quality of life (mental health and physical functioning).  Measures will include assessments of somatic symptoms (fatigue, cough, and dyspnea), depression, anxiety, QoL, coping, communication, self-efficacy for symptom control, quality of the relationship with caregiver, and caregivers’ ratings of mood, caregiver strain, caregiver self-efficacy in helping patients manage symptoms of lung disease, quality of relationship with patient, medical outcomes (death and COPD-related physician visits and hospitalisations), and patients’ health care utilization |
| Timely short-term specialised palliative care service intervention for frail older people and their family carers in primary care: Study protocol for a pilot randomised controlled trial  K. De Nooijer, L. Pivodic, N. Van Den Noortgate, P. Pype and L. Van Den Block (2021) | Frailty, life-limiting diseases, old age | RCT  Intervention: Frailty+ intervention in addition to standard care. The Frailty+ intervention is the provision of needs- and capacity-based, goal-oriented, person-centred, pro-active, and integrated palliative care, for older people and their family carers over a period of two months.  Control: standard care | Primary Outcome:  Five key symptoms that are amenable to change (i.e. breathlessness, pain, anxiety, constipation, fatigue), measured using the integrated Palliative care Outcome Scale  Secondary Outcomes:  1. Well-being of the patient: ICECAP-SCM 2. Sense of security: the SEC-P 3. Continuity of care is: NCQ 4. Views on care of the patient: IPOS-VoC 5. Coping strategies used by the patient: brief COPE 6. Sense of security of main family carer: SEC-R 7. Carer support needs: CSNAT |
| Evaluation of the Effect of Training in the Situation Background Assessment Recommendation (SBAR) Tool on the Feeling of Self-efficacy of Carers of Patients With Lung Cancer in the Face of a Situation of Simulated Respiratory Distress. (SAECURE)  NCT05839353 | Caregivers of lung cancer patients | Observational study  Intervention: simulated training by Serious Game (SG) for the use of the SBAR tool associated with the usual instructions issued in routine care, on the management of dyspnea | Effect of training in the SBAR tool by serious game on the feeling of self-efficacy of carers of patients with lung cancer in the face of a situation of simulated respiratory distress: Generalized Self-Efficacy (GSE)  Effect of training in the SBAR tool by serious game on anxiety: Hospital Anxiety and Depression Scale (HADS)  Effect of training in the SBAR tool by serious game on stress: Perceived Stress Scale-10 (PSS 10) |
| SINFONIA study protocol: a phase II/III randomised controlled trial examining benefits of guided online group singing in people with chronic obstructive pulmonary disease and interstitial lung disease and their carers  Natasha Smallwood, Amy Pascoe, Sara Vogrin, Jennifer Philip | COPD or ILD, dyspnea score (mMRC) of two or more  Carer optional enrolled | parallel, double-arm, randomised, blinded-analysis, mixed-methods phase II/III trial of guided, online group singing that will be conducted over 24 months  Intervention: one 90 min, guided, online, group singing session per week for 12 weeks and  Control: routine care | Feasibility and acceptability: Number of participants enrolled, randomised, and who complete the program. Completion is defined as attending 8 of 12 singing sessions or completing the week 12 assessment for control arm participants  Quality of life: SF-36  Anxiety and depression: HADS  Breathlessness: D-12  Mastery of breathing: CRQ-SAS  Exercise tolerance: 6MWT  Loneliness: UCLA-3  Health care utilization: Self-reported |
| Understanding living with tracheostomy ventilation for motor neuron disease and the implications for quality of life: a qualitative study protocol  Eleanor Wilson, Nicola Turner, Christina Faull, Jonathan Palmer, Martin R Turner, Scott Davidson | people living with motor neuron disease  living with tracheostomy ventilation  and caregivers | Interview Study with patients, family caregivers and health care professionals | (1) Patient focused case studies (n=6) including plwMND, family members and HCPs to focus on experiences and tasks of daily living from multiple perspectives. (2) Interviews with plwMND (n=10), family members, including bereaved family members (n=10) and HCPs (n=20) on broader experiences and issues relating to use of TV, such as ethical considerations and decision making. |

**Supplement IV: Quality Assessment (MMAT)**

| **Author, year** | **Screening** | | **Study type** | | | | | **Sum*** |
| --- | --- | --- | --- | --- | --- | --- | --- | --- |
| **Qualitative studies** | | | | | | | | |
|  | **S1** | **S2** | **1.1** | **1.2** | **1.3** | **1.4** | **1.5** |  |
| **Aasbo 2017** | Yes | Yes | Yes | Yes | Yes | Yes | No | 80% |
| **Bailey 2004** | Yes | Yes | Can´t tell | Yes | Can´t tell | No | Can´t tell | 20% |
| **Bergs 2002** | Yes | Yes | Yes | Yes | No | Yes | No | 60% |
| **Booth 2003** | Yes | Yes | Yes | Yes | Yes | Yes | Yes | 100% |
| **Booth 2006** | Yes | Yes | Yes | Yes | Yes | Can´t tell | Yes | 80% |
| **Clancy 2009** | Yes | Yes | Yes | Yes | No | No | No | 40% |
| **Collier 2017** | Yes | Yes | Yes | Yes | Yes | Yes | Yes | 100% |
| **Ek 2011** | Yes | Yes | Yes | Can´t tell | Yes | Yes | No | 60% |
| **Farquhar 2017** | Yes | Yes | Yes | Yes | Yes | Yes | Yes | 100% |
| **Ferreira 2020** | Yes | Yes | Yes | Yes | Yes | Yes | Yes | 100% |
| **Ferreira 2022** | Yes | Yes | Yes | Yes | Yes | Yes | Yes | 100% |
| **Ferreira F. 2020** | Can´t tell | Can´t tell | Can´t tell | No | Can´t tell | Can´t tell | Can´t tell | 0% |
| **Gysels 2009** | Yes | Yes | No | Yes | Yes | Yes | No | 60% |
| **Hynes 2012** | Yes | Yes | Yes | Yes | Yes | No | No | 80% |
| **Moody 2004** | Yes | Yes | Yes | Yes | Can´t tell | No | Can´t tell | 40% |
| **Pooler 2018** | Yes | Yes | Yes | Yes | Yes | Yes | Yes | 100% |
| **Reitzel 2022** | Yes | Yes | Yes | Yes | Yes | No | Yes | 80% |
| **Rocker 2012** | Yes | Yes | Yes | Yes | Yes | Yes | Yes | 100% |
| **Schunk 2019** | Yes | Yes | Yes | Yes | Yes | Yes | Yes | 100% |
| **Sigurgeirsdottir 2020** | Yes | Yes | Yes | Yes | Yes | Can´t tell | Yes | 80% |
| **Randomised studies** | | | | | | | | |
|  | **S1** | **S2** | **2.1** | **2.2** | **2.3** | **2.4** | **2.5** |  |
| **Choratas 2020** | Yes | Can´t tell | No | Can´t tell | Can´t tell | No | Can´t tell | 0% |
| **Given 2006** | Yes | Yes | Can´t tell | Yes | No | Can´t tell | Can´t tell | 20% |
| **Schunk 2021** | Yes | Yes | No | Can´t tell | Yes | No | Yes | 40% |
| **Quantitative non-randomised studies** | | | | | | | | |
|  | **S1** | **S2** | **3.1** | **3.2** | **3.3** | **3.4** | **3.5** |  |
| **Al-Gamal 2013** | Yes | Yes | No | Yes | Yes | Can´t tell | Yes | 60% |
| **Bernabeu-Mora 2016** | Yes | Yes | No | Yes | Yes | Yes | Can´t tell | 60% |
| **Freeman 2016** | Yes | Yes | Yes | Yes | Yes | No | Yes | 60% |
| **Krug 2016** | Yes | Yes | No | Yes | No | Yes | Yes | 60% |
| **Mi 2017** | Yes | Yes | Yes | Yes | Yes | No | Can´t tell | 60% |
| **Mi 2018** | Yes | Yes | Yes | Yes | Yes | No | Can´t tell | 60% |
| **Seow 2021** | Yes | Yes | Yes | Yes | No | Yes | Can´t tell | 60 & |
| **Winger 2018** | Yes | Yes | Yes | Yes | Yes | Yes | Yes | 100% |
| **Smallwood 2019** | Yes | Yes | Yes | Yes | Yes | Yes | Yes | 100% |
| **Grosbois 2022** | Yes | Yes | Yes | Yes | Yes | No | Yes | 80% |
| **Yi 2022** | Yes | Yes | Yes | Yes | Yes | Yes | Yes | 100% |
| **Quantitative descriptive studies** | | | | | | | | |
|  |  |  | **4.1** | **4.2** | **4.3** | **4.4** | **4.5** |  |
| **Brizzi 2021** | Yes | Yes | Yes | No | No | No | Yes | 20% |
| **Celik 2022** | Yes | Yes | Yes | Yes | Yes | No | Yes | 80% |
| **Fernández-García 2021** | Yes | Yes | Can´t tell | Can´t tell | Yes | Yes | Yes | 60% |
| **Granados-Santiago 2023** | Yes | Yes | No | No | Yes | Can´t tell | Can´t tell | 20% |
| **Jesus 2022** | Yes | Yes | Yes | Yes | Yes | Can´t tell | Yes | 80% |
| **Lyons 2020** | Yes | Yes | Yes | Can´t tell | Yes | No | Yes | 60% |
| **Malik 2013** | Yes | Yes | Yes | Yes | Yes | Yes | Yes | 100% |
| **Moody 2003** | Yes | Yes | Yes | Yes | Yes | Yes | Yes | 100% |
| **Oechsle 2013** | Yes | Yes | Yes | Yes | Yes | No | Yes | 80% |
| **Tang 2011** | Yes | Yes | Can´t tell | Can´t tell | Yes | No | Yes | 40% |
| **Takao 2023** | Yes | Yes | Yes | Yes | No | Can´t tell | No | 20% |
| **Yamamoto 2021** | Yes | Yes | Yes | Can´t tell | Yes | Can´t tell | No | 40% |
| **Manivannan 2022** | Yes | Yes | Can´t tell | Yes | Yes | Can´t tell | Yes | 60% |
| **Mixed methods studies** | | | | | | | | |
|  |  |  | **5.1** | **5.2** | **5.3** | **5.4** | **5.5** |  |
| **Farquhar 2014** | Yes | Yes | Yes | No | Yes | Yes | Yes | 80% |
| **Farquhar 2016** | Yes | Yes | Yes | Yes | Yes | No | Yes | 80% |
| **Schloesser 2022** | Yes | Yes | Can´t tell | Can´t tell | Yes | Yes | Can´t tell | 40% |
| **Swan 2019** | Yes | Yes | Yes | Yes | No | No | Yes | 60% |
| **Hutchinson 2022** | Yes | Yes | Yes | Yes | Yes | Yes | Yes | 100% |
| **Screening questions (for all studies)**  S1: Are there clear research questions?  S2: Do the collected data allow to address the research questions?  **Qualitative studies**  1.1. Is the qualitative approach appropriate to answer the research question?  1.2. Are the qualitative data collection methods adequate to address the research question?  1.3. Are the findings adequately derived from the data?  1.4. Is the interpretation of results sufficiently substantiated by data?  1.5. Is there coherence between qualitative data sources, collection, analysis and interpretation?  **Quantitative randomised controlled trials**  2.1. Is randomisation appropriately performed?  2.2. Are the groups comparable at baseline?  2.3. Are there complete outcome data?  2.4. Are outcome assessors blinded to the intervention provided?  2.5. Did the participants adhere to the assigned intervention?  **Quantitative non-randomised studies**  3.1. Are the participants’ representative of the target population?  3.2. Are measurements appropriate regarding both the outcome and intervention (or exposure)?  3.3. Are there complete outcome data?  3.4. Are the confounders accounted for in the design and analysis?  3.5. During the study period, is the intervention administered (or exposure occurred) as intended?  **Quantitative descriptive studies**  4.1. Is the sampling strategy relevant to address the research question?  4.2. Is the sample representative of the target population?  4.3. Are the measurements appropriate?  4.4. Is the risk of nonresponse bias low?  4.5. Is the statistical analysis appropriate to answer the research question?  **Mixed methods studies**  5.1. Is there an adequate rationale for using a mixed methods design to address the research question?  5.2. Are the different components of the study effectively integrated to answer the research question?  5.3. Are the outputs of the integration of qualitative and quantitative components adequately interpreted?  5.4. Are divergences and inconsistencies between quantitative and qualitative results adequately addressed?  5.5. Do the different components of the study adhere to the quality criteria of each tradition of the methods involved?  *Sum:”Can´t tell” statements are calculated as a “no”. | | | | | | | | |

**Supplement V: Quantitative Studies**

| **Author, year, (country)** | **Type of study/Design** | **carer characteristics** | **Measures** | **Results** |
| --- | --- | --- | --- | --- |
| **Al-Gamal et al. 2013**  **(Jordanian)** | descriptive cross-sectional study | 67 COPD carers  Age: mean (SD) 51.33 (13.97)  40 males, 27 females | *Breathlessness:* Dyspnea 12 Scale (D-12)  *Psychological distress:* Hospital Anxiety and Depression Scale (HADS) | *Relationship between spouse psychological distress (anxiety and depression) and breathlessness severity:* (r = 0.307, P < 0.05; r = 0.286, P < 0.05). |
| **Bernabeu-Mora et al. 2016 (Spain)** | prospective descriptive study  Follow-Up: 3 months | N: 87 COPD carers after 3 months 84 (2 died, 1 patient died)  Age: 62 mean  Gender: 78 females, 9 males | *Carers Depression:* Subscale of the Goldberg test  *Breathlessness:* mMRC Dyspnea Scale | Multivariate models of predictors of carer depression: spousal caregiving, having breathlessness, and severe airflow limitation were the strongest independent predictors of carer depression at the time of hospitalisation of COPD. |
| **Brizzi et al. 2020 (USA)** | Cross-sectional study Survey (online) | N: 637 ALS (444 of living patient)  Age: 55-64 years  Gender: 79% female | Categorised: carers of living patients with ALS (C-LPALS), and carers of deceased patients with ALS (C-DPALS) | Group of C-DPALS were more worried about choking than ALS Patients   - C-LPALS sign. more worried than C-DPALS (45% vs. 31%, p=.025) - C-DPALS sign. more worried than PALS (45% vs. 31%, p=.007) |
| **Celik et al. 2022 (Turkey)** | Cross-sectional Study | N: 98 cancer carers  Age: mean 45 years  Gender: 65 female, 33 males | *Caregiver Burden:* Zarit Burden Interview (ZBI),  *Breathlessness:* Edmonton Symptom Assessment Scale (ESAS) | Result for breathlessness and carer burden:  Breathlessness mean scores were significantly higher in those with high carer burden (p < .05).   - 64 carers with high burden - 34 carers with low burden   Mean score for breathlessness with ESAS:   - In high carer burden group: 5 - In low carer burden group: 3 |
| **Fernández-García et al. 2021**  **(Spain)** | Cross-sectional Study | 91 COPD carers  Age: NA  Gender: 90 (90.9%) women | *Caregiver:* Zarit Burden Interview (ZBI)  *Breathlessness:* mMRC dyspnea scale | ZBI: mean score 51.4 ± 14.2   - mild–moderate burden (score 46-55 points) 34 (37.4%) cases - Intense burden (score ≥ 56) in 29 cases (31.9%) - 25 carer (27.5%) did not report any overburden   *Logistic regression analysis:* to evaluate the variables that were independently related to carer burden were:   - previous use of social resources [OR = 8.1 (95% CI = 1.03–69.9); p = 0.04] - degree of mMRC dyspnea 3–4 [OR =4.7 (95% CI = 1.7–13.2); p = 0.003] - and two or more admissions for acute exacerbation in the previous year [OR = 4.5 (95% CI = 1.7–13.2); p = 0.003].   Of the informal carers of patients who had presented two or more of these variables, 92.3% perceived an overburden. |
| **Freeman et al. 2016**  **(Canada)** | cross-sectional study | NA | *Level of Dyspnea:* own classification ((a) no dyspnea up to (d) present at rest)  *Overall Distress:* Depression Rating Scale (DRS)  *Distress:* ((a) IC unable to continue care activities, (b) IC expressed feelings of distress, anger or depression, (c) IC reports feeling overwhelmed by ilness | - 25.2% of carers exhibited signs of distress - Carers of patients with breathlessness were more likely to exhibit distress (p =.005). - Less than 10% of carers reported feeling unable to continue their caregiving activities, while 14.7% reported feelings of distress, anger, or depression and 18.5% reported feeling overwhelmed by the person’s illness. |
| **Grenados-Santiago 2023**  **(Spain)** | Cross-sectional study | N: 70 COPD carers  Two groups based on carers’ burden (35/35)  Age: with burden: 70y (SD 3.57)  Without burden: 47.5 (SD 32.9)  Gender: predominantly females | Psychosocial distress: HADS  Carers Burden: ZBI  Breathlessness: mBorg Scale | Anxiety and depression higher in CG Burden group: HADS: 24.83 SD 10.11 vs. 15.6 SD 8.74  Area under the Curve analysis: the cut-off point for breathlessness level related to patients functionality in ADL was 19 and AUC was 0.722 (sensitivity 0.83 and specificity 0.93). |
| **Grosbois et al. 2022 (France)** | Retrospective study | N: 138 COPD carers (Program completed: 113)  Age: mean 60.3 (SD 14.8)  Gender: 41 males | Intervention: 8 Weeks home-based pulmonary rehabilitation (PR) program, with weekly supervised sessions, physical training, education, self-management strategies. IC helped to design patients action pan.  If IC experienced similar burden like patients: interventional parts were also applied to them: educational support, behavioral therapies, self-management strategies, to meet their needs.  Measures:  *Caregivers Burden:* ZBI  Anxiety and depression: HADS  Fatigue Assessment Scale  Breathlessness: mMRC | Delta (M2 – M0) after PR:   - ZBI Score: – 2.5 (SD 11.4); p 0.024 - Anxiety symptoms: - 0.9 (SD 3.5); p 0.006 - Depressive symptoms: - 0.6 (SD 3.1); p 0.047 - FAS score: - 1.4 (SD 6.6); p 0.026   Association between mMRC and changes in IC burden, anxiety and depressive symptoms and general fatique after PR:  Higher baseline mMRC score were associated with decrease in IC burden after PR. (Estimate – 2.51 (SE 1.00) with p-value 0.014) for mMRC 1-point increase  Other Changes were not significant. |
| **Jesus 2022 (Brazil)** | Cross-sectional Study | N: 54 carers of patients with LTOT  Age: mean 73.4 SD: 9.8  Gender: 36 females | Caregiver Burden Inventory (CBI)  Carers‘ quality of life: EQ-5D European Quality of life index  Breathlessness: mMRC | Univariate linear regression: increased CG Burden total score was correlated with higher breathlessness (P = 0.006) r. = 0.369  CBI dimension:  Time-dependence burden: correlated with higher limitation due to breathlessness (P = 0.01) r. = 0.335  Development Burden: correlated with higher limitation due to breathlessness (P = 0.02) r. 0.309  Increased physical and emotional burden correlated with higher limitation due to breathlessness (p = 0.007 and 0.01) r. = 0.363 and r. = 0.333  Social burden r. = 0.162, P = 0.24 (not significant)  Increased Total score of CG Burden was correlated with worse quality of life for carers |
| **Krug et al. 2016 (Germany)** | Secondary analysis of a prospective observational cohort study  Follow-Up: 6 months | N: 58 carers of patients on palliative station (dying within six month) 36 carers lost to follow-up  Age: 57.1 (SD 15.3)  Gender: 45 females, 13 males | Patients and family carers fill in on monthly intervals:  *Patients quality of life (breathlessness):* EORTC Quality of Life Questionnaire Core 15 Palliative (QLQ-C15-PAL)  *Carer burden:* short form of the Burden Scale for Family Caregivers (BSFC) | Positive correlation between: change of carer burden and change of breathlessness: ((t2-t1) reg. coefficient: 0.05, 95% CL [0.01, 0.09]; .p-value 03)  Single regression analyses:  influences on the development of carer burden between t3 and t1 of the following difference variables: breathlessness between t2 and t1, breathlessness between t3 and t1  Stepwise regression model: the difference in emotional functioning and the difference in breathlessness showed an influence on the development of carer burden (explained variance of 19.3%). |
| **Lyons et al. 2020 (USA)** | secondary data analysis of a longitudinal study  Follow-Up: 12 months | N: At baseline: 109,  at 12 months: 68 cancer carers  Age: mean 60.5 years (SD=14.1)  74% female | *Psychosocial distress:* Short Form Health Survey 2 (SF-36v2)  *Breathlessness*: adapted version of the UCSD Shortness of Breath Questionnaire (SOBQ)  *Quality of patient and carer-relationship:* Mutuality Scale | Greater incongruence in patient breathlessness (between carers and patient rating) was significantly associated with worse physical health for care partners: mean health score over 12 month for breathlessness:   - Patients: 32.30 (4.26) - Carer: 55.31 (4.51) - With *p* < .001.   Care partners, had significantly worse mental health than patients: χ^2^(df 1, *n* = 109 = 10.65, *p* < .01).  Care partners reported significantly poorer mental health when they were women, cared for younger patients, and cared for patients who were women. (in relation to pain and breathlessness) |
| **Malik et al. 2013**  **(UK)** | Descriptive cross-sectional study | N: 101 (50 lung cancer and 51 hearth failure) carers (  Age: mean (SD) years  HF: 65.8 (12.7)  LC: 59.9 (12.8)  Gender: women (82% of HF and 74% of LC carers) | *Caregiver Burden*: Zarit Burden Interview Short Form 12 (ZBI-12),  *Psychosocial distress:* Hospital Anxiety and Depression Scale (HADS), Pittsburgh Sleep Quality Inventory (PSQI), Short Form-36 (SF-36)  *Coping style:* problem-focused, emotion-focused, dysfunctional-focused  Positive caring experiences scale  *Breathlessness:* modified Borg Breathlessness Scale | **Carer outcomes:**  *ZBI-12 Burden:*  Mean burden scores were similar in both career groups, and severe burden (score of >16 on the ZBI-12) were reported from:   - LC Carer 30% (95% CI = 17–43%) - HF Carer 19% (95% CI = 8–30%)   *Positive caring experiences:*  Both groups of carers rated similar positive aspects and rewards from their caring experiences (mean score of 21.5 on the positivity scale for HF and 22.0 for LC carer).  *HADS:* Overall anxiety scores were higher than depression scores in both groups.  *HADS-A:*   - LC Carer mean 8.2 (SD 4.4) - HF Carer mean 7.7 (SD 4.1)   *HADS-D:*   - LC Carer mean 5.1 (SD 3.6) - HF Carer mean 4.6 (SD 3.5)   *Quality of life (SF-36):* similar on both groups.  *Sleep (PSQI):*   - LC Carer mean 7.8 (3.7) - HF Carer mean 8.0 (SD 4.1)   *Coping strategies:* no differences between groups. The most common coping strategy employed was ‘acceptance’, with over 90% of carers from both groups.  Factors associated with carer burden:   - No association between burden and patients’ diagnosis or severity of breathlessness - Significant association between burden and   - Carer depression   - Carer anxiety   - Worse quality of life   - Poor quality of patient care   - Patients well-being   - Carer sleep   - Carer age   Factors associated with positive caring experiences:   - Significant associations between positive caring experiences and patient’s severity of breathlessness, carers´ mental health-related quality of life, carers´ depression and anxiety.   **Summary:** Higher burden was associated with poorer ‘quality of patient care’ and worse carer psychological health (R^2^ = 0.37, F = 12.2, p = 0.01).  Carer depression and looking after more breathless patients were associated with fewer positive caring experiences (R^2^ = 0.15, F = 4.4, p = 0.04). |
| **Manivannan 2023 (India)** | Cross-sectional study | N: 220 cancer carers  Age: mean 45.5 (SD 14)  Gender: 119 females | Caregiver Burden: ZBI  Carers Quality of life: WHO QOL BREF  Patients symptoms and Quality of life: EORTEC QLQ C15PAL | Correlations:  Small sig. positive between EORTEC QLQ C15 PAL symptom score (breathlessness, insomnia etc.) and ZBI: Values between breathlessness and ZBI: Spermans correlation Rho 0.154 (95% Cl 0.018, 0.284; p-value 0.022) |
| **Mi 2017 (UK)** | Cross-sectional study | N: 113 COPD carers  Age: mean 64.2 ± 14.5 years  72.6% females | *Psychological distress:* Hospital Anxiety and Depression Scale (HADS)  *Breathlessness:* Patient disease specific health related quality of life: CRQ (subdomains with breathlessness) | The prevalence of symptoms of anxiety and depression was 46.4% (n=52) and 42.9% (n=48) in patients, and 46% (n=52) and 23% (n=26) in carers, respectively.  No significant association for patient’s breathlessness and carers anxiety or depression. |
| **Mi 2018 (UK)** | Cross sectional study | N: 117 COPD carers  Age: mean 64.16 ±14.5  27.4% males | *Unmet Needs:* 14-item Carers Support Needs Assessment Tool (CSNAT)  *Psychological distress:* Hospital Anxiety and Depression Scale (HADS)  *Breathlessness:* MRC dyspnea scale | The most bothersome symptoms were breathlessness and fatigue, followed by anxiety and depression. There was no significant difference between mean patient and carer scores for any symptom at the group level.  Multivariate analysis: the association between unmet support needs in more direct carers support domains and greater estimation of breathlessness by carers remained when adjusted for patient and carers age and sex (odds ratio 1.250, 95% CI 1.031–1.516), as did younger patient age and greater patient estimation of depression (odds ratio 1.090, 95% CI 1.018–1.167). |
| **Moody 2003 (US)** | descriptive, cross-sectional study | N: 163 lung cancer patient/carer dyads  Age: mean 61.86 (SD 14.46)  Gender: NA | *Distress:* Memorial Symptom Assessment Scale (MSAS) symptom occurrence and the distress associated with these symptoms.  *Breathlessness:* 11-point Dyspnea Graphic Rating Intensity Scale (DGRIS)  *Quality of life:* The Hospice Quality of Life Index (HQLI)  *Carers Mastery (Self-Efficacy):* was assessed by a six-item summated rating scale with a five-point scale ranging from "None (0)" to "A Great Deal (5)". | **For carers: mean (SD)**  Mastery Level (Range 10 to 42): 25.02 (4.84)  MSAS (Range 10 to 112): 25.41 (12.61)  Health Related Quality of Life (Range 11 to 116): 55.06 (22.10)  Rating of Patients' breathlessness (Range 2 to 9) 4.39 (2.93)  Factors influencing Carers' Quality of Life (Mastery measurement):  stepwise multiple regression to determine if the carers´ perceived levels of mastery, symptom distress, age, educational level, and the patient's breathlessness intensity were significantly related to their perceived quality of life (R^2^ = .40, p = .02). |
| **Oechsle et al. 2013 (Germany)** | cross-sectional Study | 33 cancer carers  Age: NA  Gender: 22 females, 11 males | *Anxiety:* PHQ-9 and GAD-7 German version  *Depression:* PHQ-9 German version  *Breathlessness:* Memorial Assessment Scale (MSAS) German version | 55% of male carers and 36% of female carers showed moderate or severe anxiety; 36% of male carers and 14% of female carers had moderate or severe depression. Carers’ anxiety was associated with a discrepancy in the patients’ symptom evaluation for shortness of breath (p < 0.05); nausea (p < 0.05); and frequency, intensity, and distress due to anxiety (p < 0.01).  *Correlation between Carers ‘anxiety and Depression and total shortness of breath (Frequency, Intensity, Distress, Treatment)*   - Total with Anxiety r = .26 - Total with Depression r = .37   *Significant correlations between carers’ depression and*  *their evaluation of patients’ symptoms:*   - frequency of breathlessness (r = 0.38; p = 0.030), - intensity of breathlessness (r = 0.37; p = 0.037) - patients distress level due to breathlessness (r = 0.39; p = 0.029) - total score of shortness of breath (r = 0.37; p = 0.036)   Lower carers’ anxiety and depression were not significantly associated with an underestimation of symptoms.  Trend towards a higher risk of overestimation of symptoms in carers with higher levels of anxiety (r = 0.32) (p = 0.07) and depression (r = 0.33) ( p = 0.06). |
| **Seow et al. 2021**  **(Canada)** | Retrospective observational cohort study  Data from the interRAI Home Care Reporting System (RAI-CH). | NA | *Distress (anger, depression):* measured as a single item (yes/no)  *Breathlessness:* Yes/no | Carers distress was only measured in the home care cohort. Among those who reported carer distress, 28.3% patients had clinician-reported breathlessness documented.  Multivariate regression: the presence of breathlessness was associated with a greater odds of carer distress (OR=1.19, 95% CL: 1.18-1.20), controlling for other covariates. |
| **Smallwood et al. 2019**  **(Australia)** | Cross-sectional study | N: 24 (60.0%) responses from 40 eligible carers included 11 (45.8%) carers completed the questionnaire as interviews in clinic and 13 (54.2%) carers returned the survey by post.  Patients from Advanced Lung Disease Service | The Advanced Lung Disease Service (ALDS) is a partnership between respiratory and palliative medicine and focuses on active symptom management, individualized patient, and carer education (including providing written resources), and advance care planning.  Survey questions focused on four main themes:  (1) ALDS hospital clinic (usefulness of clinic visits, symptom management, health information discussions and waiting time to be seen)  (2) ALDS nursing support service (types of telephone support accessed and home visits)  (3) General views and overall opinion (including confidence in the service, feeling heard and respected, having enough time and opportunities to discuss important aspects of care, and valued elements of the service)  (4) Areas for future improvement. | (1) ALDS helped with symptom support in case of breathlessness: all carers said yes (100%).  66.7% of the carers did not request any additional information in addition to the topics discussed (Underlying condition, severity of condition, breathlessness, managing a breathlessness crisis, breathlessness self-management techniques, oxygen therapy, exercise/pulmonary rehabilitation, advanced care planning, Medical power of attorney), only 7 (29.2%) carers requesting these resources.  (2) 12 carers (50.0%) recalled using the telephone service directed by the ALDS nurse. The telephone support services were considered helpful by patients and carers who used them. One-third of family carers (33.3%) recalled a home visit from the ALDS respiratory specialist, with most participants (95.6% of patients and 87.8% of carers) finding this helpful.  (3) No specific carer results.  (4) 20 (83.3%) carers did not want to see additional healthcare professionals during their ALDS clinic visits.  Wished a visit by following professionals:   - Physiotherapist or Occupational therapist 6 (37.5) - Psychologist 1 (4.17%) - Palliative and supportive care nurse 0   *What aspects of our service are important to you or your relative? (Carer Results)*   - Continuity of care 23 (95.8) - Long term care 21 (87.5%) - Urgent Review 16 (66.7%) - Nurse specialist telephone support 11 (45.8%) - Nurse specialist visits the home 10 (41.7%) - Extended consultations 13 (54.2%) - Afternoon appointments 9 (37.5%) - Palliative and supportive care doctor 10 (41.7%) |
| **Takao 2023**  **(Japan)** | Cross-sectional study | N: 670 bereaved family members: Subgroup of 86 carers of terminal cancer and dementia patients and 587 without perceived dementia  Age: NA  Gender: NA | Caregiver Consequence Inventory (CCI) was used to measure carers´ burden  Breathlessness: participants reported the severity of the symptom one week before death on a scale ranging from 0 (not at all) to 4 (very acute). | The carers´ burden was statistically significantly higher (3.61 ± 1.58 vs 3.22 ± 1.47; p < 0.036) among carers of terminal cancer patients with dementia.  Breathlessness (odds ratio, 1.67, CI 1.10–2.55, P-Wert: 0.015 ) were contributing factors for carer burden when perceived dementia, and OR 1.14, Cl 0.95–1.37, P-Wert: 0.153 for carer burden without perceived dementia |
| **Tang et al. 2011**  **(China)** | cross-sectional study | N: 112 Silicosis carers  Age: mean (SD): 57.4 (11.3) y  Gender: 2 males, 110 females | *Carer:*  Caregiving Burden Scale (CBS).  Carers’ QOL with Short Form-36 (SF-36). Composed of a physical component (PCS) and a mental component (MCS).  Carers’ cognitive function was assessed with the MMSE.  The availability of family support: defined as the presence or absence (yes/no)  Patients:  The Medical Research Council Dyspnea (MRCD) score quantified the impact of breathlessness | **Correlates of Carers´ Burden**:  Carers´ burden was significantly correlated with severity of breathlessness (r = .359, P < .0001),  **Correlates of Physical QOL**   - Carers SF-36 scores: PCS: Mean (SD) 54.4 (10.0) - Univariate Analysis: Poorer PCS was significantly correlated with patients’ severity of breathlessness (r = .223, P = .019), - Multivariate Analysis: Two patient-related variables (severity of breathlessness and coexisting diseases) and two carer variables (cognitive function and depressive symptoms) were entered into the PCS regression model, where patients’ severity of coexisting diseases and carers’ depressive symptoms proved to be significant correlates of caregiving burden, explaining 34% of the variance.   **Correlates of Mental QOL**   - Carers SF-36 scores: MCS: Mean (SD) 51.8 (10.1) - Univariate Analysis: MCS was significantly correlated with severity of breathlessness (r = .299, P = .001), - Multivariate Analysis: Patients’ depressive symptoms and severity of breathlessness and carers’ depressive symptoms were significant correlates of the MCS, explaining 56% of the variance |
| **Winger et al. 2018**  **(USA)** | A within-subject design to analyze data from participants who were randomised to TSM in a RCT | N: 51 lung cancer carers  Age: mean (SD) 56.33 (14.09)  Gender:  Male 14 (27.45%)  Female 37 (72.55%) | *Telephone Symptom Management (TSM)* For patients and carers participated in the intervention concurrently via speakerphone. Each participant received a notebook with handouts and other study materials. 4 secessions of different coping strategies.  Breathlessness: item assessing distress related to breathlessness from the Memorial Symptom Assessment Scale  Patients’ and carers’ psychological distress was assessed using the Patient Health Questionnaire and the Generalized Anxiety Disorders scale. | All the regression models explained significant variability in symptoms at six weeks after the intervention, including 40% of patients’ pain interference, 44% of patients’ fatigue interference, 38% of patients’ distress related to breathlessness, 49% of patients’ psychological distress, and 64% of carers’ psychological distress.  Greater practice of guided imagery demonstrated a significant, moderate association with less psychological distress (β = .30, P = 0.01) for carers.  Greater practice of problem solving demonstrated a significant, moderate association with higher distress related to breathlessness (β = 0.56, P = 0.01) and a non-significant, moderate association with higher psychological distress (β = 0.36, P = 0.08) for patients.  Emotion-focused coping: Carers  Mean (SD): 4.93 (4.87)  Range: 0 – 19 |
| **Yamamoto et al. 2021**  **(Japan)** | cross-sectional survey  This study was part of the fourth Japan Hospice and Palliative Care Evaluation (J-HOPE4) study, which is a quality improvement project. | Analyzed 231 out of 533 carers of dying cancer patients  Age: mean (SD) 63.9 (12.5)  Gender: 76 males, 148 females | *Family-reported use of care strategies to relieve terminal dyspnea:* with self-developed 19 items questionnaire  *Satisfaction with care provided for terminal dyspnea*: by asking questions  *Family-perceived intensity of terminal dyspnea and use of oxygen for terminal dyspnea:* respond options were severe breathlessness, moderate breathlessness, and mild breathlessness | **Family perceived care for terminal breathlessness:**  Factor Score in Total: Agree 3; slightly agree, 2; and slightly disagree and disagree, 1. N = Sum of the responses to agree and slightly agree.  *Factor 1:* Dedicated and compassionate care (Mean = 2.3, SD = 0.6)  *Factor 2:* Practical and physical care (Mean = 2.0, SD = 0.6)  *Factor 3:* Care for family members (Mean = 2.0, SD = 0.7)   - Helping the family easily to understand the patient’s cause of breathlessness: Total n=159 (68.8%) - Managing breathlessness while respecting families’ preferences: Total n=150 (64.9%) - Promoting family involvement in the process of care: Total n=141 (61%) - Listening to families’ anxiety and distress: Total n=135 (58.4%) |
| **Yi 2022**  **(UK)** | Economic study | N: 68 carers of COPD, ILD, lung cancer patients  Age: mean 64 years  Gender: 86% females | Discreet choice experiment: to elicit preferences and acceptability of holistic short-term multi-professional breathlessness triggered services (BSs)  Markov model for cost-effectiveness with Euro-Qol-5 and health and social care costs | Carers preferences:  Differs from patients’ preferences: X^2^ = 21.77; p < 0.04  Stated a strong preference for BS with home visits from GPs, and social worker and therapists’ involvement.  Markov model: cost-effectiveness for a 75-years old man over 5 years providing BS is cheaper (over 12 weeks) than usual care and quality of life improved. |
